# Placental malaria vaccine candidate antigen VAR2CSA displays atypical domain architecture in some *Plasmodium falciparum* strains

**DOI:** 10.1038/s42003-019-0704-z

**Published:** 2019-12-06

**Authors:** Justin Y. A. Doritchamou, Robert Morrison, Jonathan P. Renn, Jose Ribeiro, Junhui Duan, Michal Fried, Patrick E. Duffy

**Affiliations:** 1 Laboratory of Malaria Immunology & Vaccinology, National Institute of Allergy and Infectious Diseases, NIH, Bethesda, MD USA; 2 Laboratory of Malaria and Vector Research, National Institute of Allergy and Infectious Diseases, NIH, Bethesda, MD USA

**Keywords:** Parasite genetics, Malaria

## Abstract

Two vaccines based on *Plasmodium falciparum* protein VAR2CSA are currently in clinical evaluation to prevent placental malaria (PM), but a deeper understanding of *var2csa* variability could impact vaccine design. Here we identified atypical extended or truncated VAR2CSA extracellular structures and confirmed one extended structure in a Malian maternal isolate, using a novel protein fragment assembly method for RNA-seq and DNA-seq data. Extended structures included one or two additional DBL domains downstream of the conventional NTS-DBL1X-6ɛ domain structure, with closest similarity to DBLɛ in *var2csa* and non-*var2csa* genes. Overall, 4/82 isolates displayed atypical VAR2CSA structures. The maternal isolate expressing an extended VAR2CSA bound to CSA, but its recombinant VAR2CSA bound less well to CSA than VAR2CSA_NF54_ and showed lower reactivity to naturally acquired parity-dependent antibody. Our protein fragment sequence assembly approach has revealed atypical VAR2CSA domain architectures that impact antigen reactivity and function, and should inform the design of VAR2CSA-based vaccines.

## Abstract (web summary)

Justin Doritchamou et al. study VAR2CSA, a protein from *Plasmodium falciparum* currently in use in two vaccines to prevent placental malaria. They identify atypical extracellular structures that impact antigen reactivity and function, potentially impacting the design of VAR2CSA-based vaccines.

## Introduction

In malaria-endemic areas, pregnant women commonly suffer from placental malaria (PM), wherein *P. falciparum*-infected erythrocytes (IE) sequester in the placenta and often elicit an inflammatory infiltrate. Inflammatory PM is associated with severe maternal anemia, pre-term delivery, low birthweight, and perinatal mortality^1^. It is now well-established that placental sequestration results from IE that express the variant antigen VAR2CSA on their surface and bind to Chondroitin Sulfate A (CSA) in the intervillous spaces of the placenta^2,3^.

Several lines of evidence support VAR2CSA as the leading vaccine candidate to prevent PM: parasites isolated from the placenta uniformly express VAR2CSA^4,5^; levels of antibodies that inhibit IE binding to CSA, as well as antibodies that bind VAR2CSA increase with gravidity^6,7^; women who have acquired anti-adhesion antibodies are protected against PM-related adverse outcomes^7,8^; anti-adhesion antibodies have been induced by VAR2CSA vaccination in preclinical studies^9,10^; and recombinant VAR2CSA binds to CSA with nanomolar affinity^11^. Two VAR2CSA-based vaccine candidates currently in phase 1 clinical trials^12,13^ were designed to block IE binding to CSA and thereby prevent placental sequestration.

VAR2CSA, a member of the *Plasmodium falciparum* erythrocyte membrane protein 1 (PfEMP1) family, is a cysteine-rich transmembrane multidomain protein formed by six Duffy-binding-like (DBL) domains with several interdomain (ID) regions. DBL domains of PfEMP1 are classified into α to ɛ sub-classes based on multiple sequence alignment, and DBL sequences that do not fit well into existing categories are termed DBLX^14^. Exon 1 of *var2csa* encodes the extracellular region of the protein with three DBLX followed by three DBLɛ domains, and exon 2 encodes the transmembrane domain (TMD) and intracytoplasmic acidic terminal segment (ATS)^14,15^. This VAR2CSA architecture was originally defined in the prototype 3D7 clone and has been observed in all strains and clones to date, except one isolate (RAJ116) from India reported to contain an additional C-terminal DBL domain^16^. The extent of atypical VAR2CSA extracellular structures has not been well documented, in part due to difficulty with sequencing VAR2CSA in field isolates.

In an ongoing project surveying-binding phenotypes of field isolates collected from children and pregnant women in Mali, we generated RNA-seq data and analyzed *var2csa* transcripts of a maternal isolate at different timepoints during adaptation to long-term culture. Subsequently, DNA-seq and RNA-seq data from public databases were analyzed to define variation in VAR2CSA domain structure. This manuscript describes a novel approach to reconstruct protein sequences of field isolates from short Illumina reads. With this approach, we have identified atypical VAR2CSA domain architectures present in Malian and Tanzanian field isolates. We have also characterized the functional activity of a maternal isolate expressing an extended VAR2CSA.

## Results

### Isolate M200101 exhibits an extended *var2csa* sequence

**Fig. 1:**
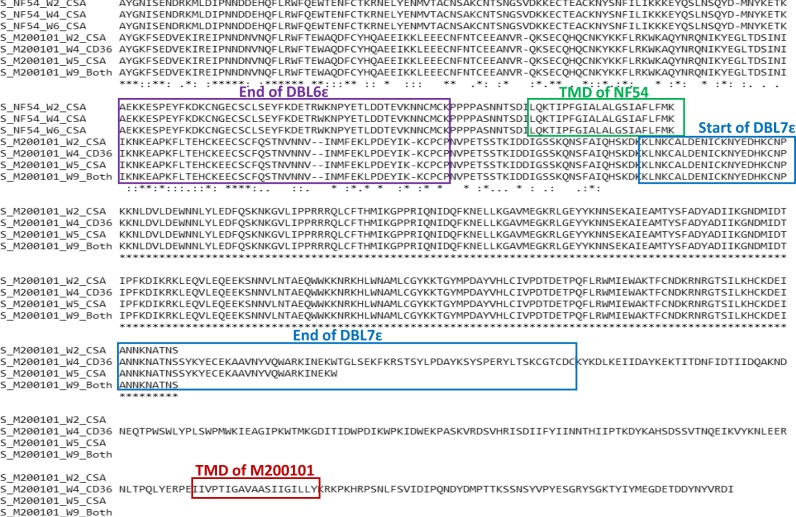
Extended DBL domain structure in VAR2CSA_M200101_. VAR2CSA sequences from the maternal isolate M200101 and NF54_CSA_ at different weeks of in vitro culture were analyzed and assembled using the consensus protein pileups tool. End of DBL6ɛ domain of VAR2CSA for both isolates is indicated in the purple box, and the transmembrane domain (TMD) motif for NF54_CSA_ is highlighted in green. The beginning and end of the DBL7ɛ domain of M200101 sequences are shown in the blue boxes. Using the TMHMM Server, v. 2.0 the predicted TMD for M200101 is indicated in the red box.

Analysis of the *var2csa* sequence generated from the maternal isolate M200101 revealed a relatively distant sequence to NF54, used as reference. Surprisingly, M200101 sequence of *var2csa* displayed an unusual DBL domain structure characterized by an extra DBL beyond the “expected” final DBL6ɛ domain of reference NF54 (Fig. 1). In addition, the analyzed samples of M200101 *var2csa* sequences at distinct culture timepoints showed that this unique *var2csa* sequence was transcribed by the isolate at all timepoints. This extended structure of M200101 *var2csa* was independently confirmed by the three different sequence analysis approaches (Supplementary Fig. 1). Based on domain sequence motifs, the extra DBL was identified as a DBLɛ. We therefore termed the identified domain downstream of the DBL6ɛ domain of “conventional” *var2csa* sequence as DBL7ɛ in M200101. *Var2csa* domains of M200101 allele showed the highest similarity scores to different reference lines as described in Table 1. Of note, the DBL7ɛ domain of M200101 *var2csa* sequence shared 100% identity (Edit_Dist = 0AA) to the DBLɛ8 domain of *DD2var28* in DD2 strain. By edit distance, the DBLɛ8 domain of *DD2var28* looks most like DBL5ɛ domains in *var2csa*.

**Table 1:** Similarity of VAR2CSA_M200101_ domains to different *Pf*EMP1 from reference strains.

Domain_ID	Query_Domain_ID* (Ref: *Pf*EMP1)	Score_per_AA	Domain cassette	UPS	Edit_Dist
NTS	NTSpam (HB3:HB3var2csaA)	4.804		UPS_E	4
DBL1X	DBLpam1 (PFCLIN:PFCLINvar72)	4.834	DC2	UPS_E	45
DBL2X	DBLpam2 (IT4:IT4var04)	4.357	DC2	UPS_E	75
ID2	CIDRpam (IGH:IGHvar41)	5.044	DC2	UPS_E	19
DBL3X	DBLpam3 (IGH:IGHvar41)	5.227	DC2	UPS_E	17
DBL4ε	DBLepam4 (IT4:IT4var04)	4.831	DC2	UPS_E	39
DBL5ε	DBLepam5 (RAJ116:RAJ116var25)	4.755	DC2	UPS_E	38
DBL6ε	DBLe10 (RAJ116:RAJ116var25)	3.506	DC2	UPS_E	109
DBL7ε	DBLe8 (DD2:DD2var28)	5.519	DC3	UPS_B	0
ATS	ATSpam1 (3D7:PFL0030c)	4.382		UPS_E	68

*ID* Identity, *AA* amino acid, *Edit_Dist* edit distance, *Domain_ID nomenclature as defined in VarDom 1.0 server is used

**Fig. 2:**
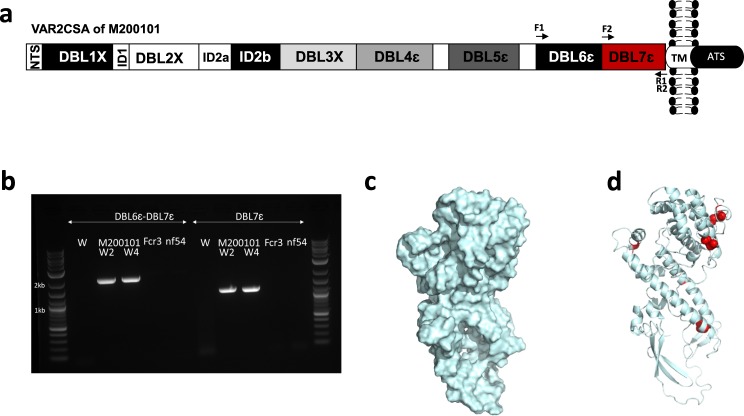
DBL7ɛ in VAR2CSA_M200101_. **a** Schematic representation of VAR2CSA sequence in isolate M200101 with positions of the designed primer pairs (F1-R1 and F2-R2) to amplify fragments encompassing DBL7ɛ. **b** Gel picture of PCR products representing DBL6ɛ-DBL7ɛ (expected size: 1999 bp) and DBL7ɛ (expected size: 1200 bp) fragments in M200101 samples collected at week 2 (w2) and week 4 (w4) of culture. FCR3 and NF54 showed no amplification of these VAR2CSA fragments. The 3D model of DBL7ɛ predicted by I-TASSER is shown as surface (**c**) and ribbon (**d**) highlighting helixes, loops, and sheets. Cysteine residues are shown in red and those involved in disulfide bonds formations are indicated with red spheres.

The predicted three-dimensional (3D) structure of the VAR2CSA_M200101_ DBL7ɛ domain was modeled (Fig. 2c, d) and the template search confirmed that the current structures of VAR2CSA DBL domains^17–20^ had the highest scores for modeling, although sequence identity between the DBL7ɛ domain and the templates varied between 15–35% (Supplementary Table 1). The predicted DBL7ɛ model was divided into helix, strand and coil, where the coil class consists of secondary structure types mostly found in loops. Twelve cysteines including six involved in disulfide bond formation were predicted by the retained model.

To prove that this extended *var2csa* sequence in M200101 can be detected in the isolate, genomic DNA was extracted from the M200101 culture sampled at 2-week intervals. We successfully amplified the DBL7ɛ as well as the double domain DBL6ɛ-DBL7ɛ fragments of *var2csa* in the M2000101 isolates, but not in FCR3 and NF54 isolates (Fig. 2a, b). In a complementary approach, Sanger sequencing was performed on the PCR products of DBL6ɛ-DBL7ɛ fragment amplification from M200101 and confirmed the DBL7ɛ sequence identified by transcriptomic data (Supplementary Data1).

### Consensus protein pileups (CPP) analysis of publicly available *var2csa* sequences

A total of 137 field samples and lab strains were analyzed by the CPP methodology (74 DNA-seq, 63 RNA-seq). Of RNA-seq samples, only 15/63 had sufficient read depth across *var2csa* for CPP to return a full-length protein sequence (Supplementary Data 2). Gene expression results suggest a minimum cutoff of about 25 RPKM is needed for *var2csa* to have enough raw reads for CPP to succeed when starting from RNA-seq data. For DNA-seq samples, lack of read coverage was not a limitation, with 73 of 74 generating full-length protein sequences. The one failing DNA-seq sample was a Tanzanian child isolate (C0111a01) exhibiting a truncated *var2csa* sequence of only 385AA containing just NTS and DBL1. Independent de novo assembly by Velvet confirmed the truncated sequence, suggesting a genomic deletion of most of *var2csa* in that isolate.

Final protein length for a typical 6-domain alleles was similar in both types of data (DNA-seq: mean 2688AA, range 2517-2851AA; RNA-seq: mean 2648AA, range 2507-2711AA). Samples possessing novel extra DBL domains were longer (mean 3168AA, range 2903-3968AA). To assess the diversity of full-length *var2csa* sequences, we calculated the edit distance of the protein as the sum of the edit distances of each domain to its best matching reference domains. Average edit-distance for DNA-seq samples was 363AA, meaning that roughly 13.5% of amino acids are altered across the extracellular region. For RNA-seq samples, average edit-distance was 383AA or 14.1% of amino acids. Interestingly, two samples had edit distance of zero (Tanzanian maternal sample M0984 is a perfect match to 3D7/NF54, and lab strain W2 is a perfect match to lab strain DD2). A phylogenetic tree of these VAR2CSA full-length proteins (Supplementary Fig. 2) showed no clear geographic clustering, suggesting that sequence variation is driven mostly by random hyper-variant mutations and not by heredity.

### Extended *var2csa* sequence is present in other field isolates

The analysis of the CPP-generated full-length VAR2CSA sequences from publicly available FASTQ datasets showed that an isolate from Mali displayed an extended DBL domain structure similar to M200101. Isolate Mali_PS122 had a DBL1X-DBL7ɛ domain organization of VAR2CSA with a DBL7ɛ sequence sharing AA identity to DBLɛ domain in *DD2var28* as seen with M200101. Furthermore, a BLAST analysis of DBL7ɛ sequence in M200101 and Mali_PS122 showed a perfect match with a partial erythrocyte membrane protein sequence (AN: AAM77858.1) that originated from a placental *P. falciparum* isolate in Kenya^21^. Another isolate (Mali_PS103) unexpectedly displayed two additional domains for a total of eight DBL domains; these two extra DBL domains had the lowest edit distance to DBL5ɛ and DBL6ɛ of *var2csa* (*DD2var06*) in DD2 (Table 2).

**Table 2:** Similarity of VAR2CSA_Mali_PS103_ domains to different *Pf*EMP1 from reference strains.

Domain_ID	Query_Domain_ID* (Ref: *Pf*EMP1)	Score_per_AA	Domain cassette	UPS	Edit_Dist
NTS	NTSpam (HB3:HB3var2csaA)	4.843		UPS_E	3
DBL1X	DBLpam1 (PFCLIN:PFCLINvar72)	4.971	DC2	UPS_E	35
DBL2X	DBLpam2 (HB3:HB3var2csaB)	4.508	DC2	UPS_E	77
ID2	CIDRpam (DD2:DD2var06)	5.039	DC2	UPS_E	22
DBL3X	DBLpam3 (IGH:IGHvar41)	5.011	DC2	UPS_E	34
DBL4ε	DBLepam4 (IGH:IGHvar41)	5.187	DC2	UPS_E	22
DBL5ε	DBLepam5 (PFCLIN:PFCLINvar72)	5.000	DC2	UPS_E	26
DBL6ε	DBLe10 (RAJ116:RAJ116var25)	3.474	DC2	UPS_E	107
DBL7ε	DBLepam5 (DD2:DD2var06)	2.761	DC2	UPS_E	120
DBL8ε	DBLe10 (DD2:DD2var06)	5.440	DC2	UPS_E	0

*ID* Identity, *AA* amino acid, *Edit_Dist* edit distance, *Domain_ID nomenclature as defined in VarDom 1.0 server is used

All extra DBL domains that we identified in VAR2CSA were DBLɛ, with those detected in the isolate Mali_PS103 appearing like a double domain DBL5ɛ-6ɛ (Table 2). A phylogenetic cluster analysis of the DBLɛ domain sequences (*n* = 148) from *var* genes according to the domain nomenclature and sequences used in VarDom 1.0 server^16^ demonstrated that DBLpam1 (DBL1X) to DBLepam4 (DBL4ɛ) domain sequences of *var2csa* were distinctly grouped (Supplementary Fig. 3). DBLepam5 (DBL5ɛ) domains also clustered together, but with one extra member (DBLe8 from *DD2var28*) that was not *var2csa*.

### M200101 isolate displays a placenta-binding phenotype

**Fig. 3:**
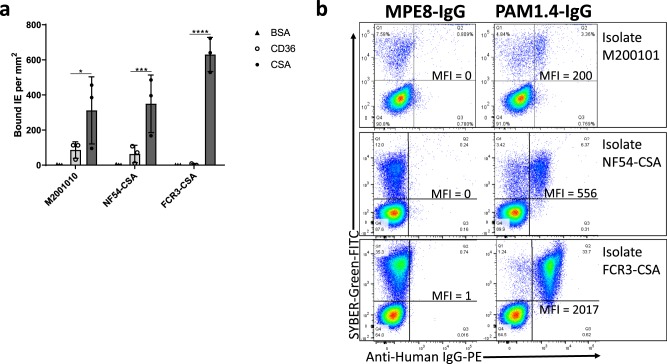
M200101 isolate surface-expresses VAR2CSA and binds to CSA. **a** Binding of M200101, NF54-CSA, and FCR3-CSA isolates to immobilized CD36 and CSA receptors was assessed by in vitro binding assay. Bovine serum albumin (BSA) was also coated on the plate for background assessment. For each receptor, the number of bound IE per mm^2^ and mean ± SD of three independent assays (*n* = 3) are shown. **b** Surface expression of VAR2CSA by the isolates was evaluated in Flow cytometry using PAM1.4, a VAR2CSA-specific monoclonal antibody, and a negative monoclonal antibody (MPE8) with no specificity to *P. falciparum*. For each antibody, the median fluorescence intensity (MFI) after subtraction of the MFI of the well with no sample, are reported.

The maternal isolate M200101 bound well to CSA but not CD36 (*p* = 0.02, Fig. 3a) and showed strong reactivity to VAR2CSA-specific human monoclonal PAM1.4 as measured by the level of PAM1.4-stained M200101 IEs (Fig. 3b). This observation suggests that the extended structure of VAR2CSA did not suppress the ability of M200101 isolate to display a placenta-binding phenotype.

**Fig. 4:**
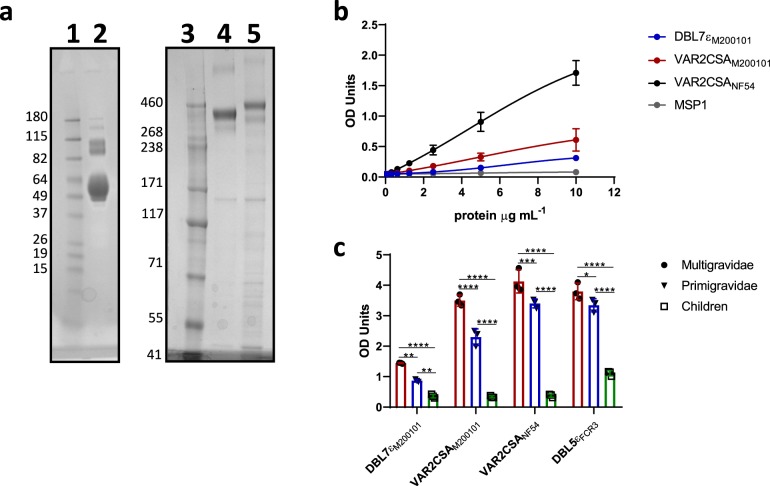
Parity-dependent reactivity of VAR2CSA recombinants from M200101 that bind to CSA. **a** VAR2CSA recombinants expressed in Expi293 cells were analyzed in SDS-PAGE gels. Ten micrograms of purified DBL7ɛ_M200101_ VAR2CSA (lane 2) was loaded on a 4–12% bis-tris gel and Coomassie stained. One microgram of purified full-length M200101 DBL1x-7ɛ (lane 5) and NF54 DBL1x-6ɛ (lane 4) were loaded on a 3–8% tris-acetate gel and Coomassie stained. Lane 1 and 3 are molecular weight markers (kDa). **b** Serial dilution of recombinant full-length VAR2CSA from M200101 (VAR2CSA_M200101_) and NF54 (VAR2CSA_NF54_), DBL7ɛ_M200101_, as well as MSP1 binding to Decorin (CSPG) was analyzed by ELISA. For each protein OD values from three biological replicates (*n* = 3) and a nonlinear regression curve fitting model were plotted. **c** Plasma pools from malaria-exposed multigravidae (red bar), primigravidae (blue bar) and children (green bar) in Mali were evaluated for antibody reactivity to VAR2CSA_M200101_ and DBL7ɛ_M200101_. Full-length VAR2CSA_NF54_ and DBL5ɛ_FCR3_ proteins were used as comparator antigens. OD values from three independent assays (*n* = 3) are shown as mean ± SD.

To further characterize the interaction between VAR2CSA_M200101_ and CSA, the mammalian-expressed full-length VAR2CSA from M200101 sequence (Fig. 4a) was evaluated for in vitro adhesion to immobilized CSPG. CSA-binding capacity of the protein was demonstrated with a concentration-dependent increase in OD values in comparison to MSP1 (merozoite surface protein 1) (used as non-VAR2CSA control antigen) (Fig. 4b). The level of this interaction to CSA appeared low compared to the binding level of full-length VAR2CSA_NF54_. Although weaker than the full-length VAR2CSA proteins, recombinant DBL7ɛ_M200101_ also bound to CSPG, suggesting that this extra DBLɛ domain protein may contain a CSA-binding site.

The enzyme-linked immunosorbent assay (ELISA) analysis of the naturally acquired antibodies from malaria-exposed individuals demonstrated that isolates expressing a similar extended VAR2CSA are circulating in Mali, where the plasma was collected. Level of IgG binding to VAR2CSA_M200101_ is higher in multigravidae compared to first-time pregnant women and children (*p* < 0.0001, Fig. 4c and Supplementary Fig. 4). A similar pattern of reactivity was observed in pregnant women and children with plasma antibodies binding to DBL7ɛ_M200101_ domain protein, although the level of reactivity was low compared to that measured with the full-length VAR2CSA_M200101_ and VAR2CSA_NF54_, as well as DBL5ε_FCR3_ domain protein.

## Discussion

Several types of *var* genes have been defined based on domain architectures, with up to seven DBL domains^14,15^. *Var2csa*, the current leading vaccine candidate against PM, is classified as type 13 based on a six DBL domain structure (NTS-DBLX_1-3_-DBLɛ_4-6_) without a typical N-terminal NTS-DBLα head structure. Many preclinical studies using allele-based sub-unit VAR2CSA DBL domain constructs have induced antibodies with limited heterologous functional activity^22,23^. Potentially, one obstacle to the design of a VAR2CSA-based vaccine with broadly neutralizing activity could be the incomplete characterization of *var2csa* diversity. In this work, we used a new approach for protein fragment sequence assembly of transcripts to identify extended *var2csa* sequences with up to eight DBL domains expressed by *P. falciparum* field isolates, highlighting the need for better characterization of *var2csa*. *P. falciparum* isolates expressing such extended VAR2CSA may affect the quaternary structure of the protein, potentially impacting the biological functions of the protein and functional epitopes.

**Fig. 5:**
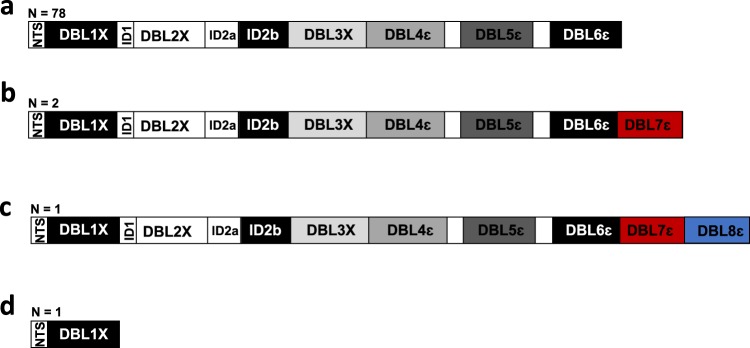
Summary of VAR2CSA DBL domain architectures. **a** Schematic of the DBL domain composition of a typical VAR2CSA with NTS-DBL1X-6ɛ is represented. Atypical structures of VAR2CSA consisting of **b** NTS-DBL1X-7ɛ and **c** NTS-DBL1X-6ɛ domain combination, and **d** the truncated *var2csa* (with NTS-DBL1X structure) are shown. The number of analyzed isolates displaying the different architecture of VAR2CSA are indicated.

To our knowledge, this report is the first to describe an 8 DBL domain combination in *var2csa*, yielding an NTS-DBL8ɛ domain structure (Fig. 5). The isolate exhibiting this domain structure of VAR2CSA was collected from Mali and the additional two C-terminal DBL domains to the NTS-DBL6ɛ structure were DBLε. Rask et al. previously noted that lab strain RAJ116 (from India), encoded *var2csa* with an extra C-terminal DBL domain^16^, leading to the NTS-DBL7ɛ domain structure. In the current work, we have also identified this NTS-DBL7ɛ domain structure in *var2csa* sequences of two field isolates from Mali. Our data suggest that these atypical structures of VAR2CSA expressed by field isolates may be prevalent in African populations and not limited to specific geographical areas. Indeed, a BLAST analysis of the DBL7ɛ VAR2CSA_M200101_ revealed identical sequence homology to a DBL sequence fragment amplified from a placental isolate in Kenya using primers based on conserved stretches of amino acids within generic DBL domains^21^. This observation suggests that these sequences of VAR2CSA with extended domain structure may be circulating in Kenya as well. In addition, extended *var2csa* sequences may not be restricted to pregnant women, as information on the donors from whom the isolates Mali_PS103 and Mali_PS122 were collected was not available to us. Thus, the distribution of *var2csa* sequences with extended domain structure should be fully explored to understand the structural diversity of *var2csa* and any relationships with the pathophysiology of PM.

Su et al.^24^ previously provided evidence of *var* gene rearrangement in parasites derived from the DD2 clones, demonstrating that DNA recombination can occur in parasites during long-term cultivation. Here, we provided evidence that this recombination event naturally occurs in *P. falciparum* field isolates with no geographical limitation. The fact that the extra C-terminal DBL domains in *var2csa*, described in this work, showed close/perfect sequence similarity with non-*var2csa var* genes (*DD2var28*) in DD2 strain was unexpected, given the origins of the isolates. While DBL domains can undergo shuffling or deletion^24^, this would require the existence of the inserted or deleted sequence in the parasite genome. The full *var* gene repertoire of these isolates with extended *var2csa* has not been studied in this work, which precludes gene similarity analysis with other parasite strains. However, *DD2var28* shares most similarity with *PFL0020c* in 3D7, which is located at the sub-telomeric locus directly adjacent to *var2csa* locus. For instance, if the gene donating its DBL domain to *var2csa* in M200101 shares the same locus as *PFL0020c* in 3D7, such proximity could facilitate recombination. Hence, further genome characterization for *var* gene repertoires in extended VAR2CSA-expressing isolates may shed additional light on the molecular mechanisms behind DBL domain extension in *var2csa* sequences.

Another novel finding from this work is the identification of a truncated *var2csa*, leading to NTS-DBL1X domain structure of protein in a child’s isolate. Among the three most conserved *var* gene families (*var1*, *var2csa*, and *var3*), a similar phenomenon has been previously reported for *var1* in isolates, including 3D7 and IT4^16^. This frequently seen truncated structure in *var1* has never been demonstrated for *var2csa*. Since the isolate with NTS-DBL1X was collected from a child, it is unclear whether functional properties of VAR2CSA may have moved to other *Pf*EMP1 variants as suggested for VAR1^16^.

An additional DBL domain(s) downstream of DBL6ɛ would result in a larger protein with a quaternary structure that differs from VAR2CSA proteins from FCR3 or 3D7, the two major alleles in the current vaccine candidates, which share NTS-DBL1X-6ɛ structures. The functional characterization of DBL7ɛ domain and NTS-DBL1X-7ɛ VAR2CSA proteins expressed by the maternal isolate M200101 indicated that this extended VAR2CSA protein can bind to CSA and is targeted by naturally acquired antibodies in parity-dependent manner. However, level of CSA-binding and antibody recognition was lower than that observed with proteins from NF54 and FCR3 variants. This finding suggests that the protein function might be reduced but not suppressed in isolates expressing extended VAR2CSA. Whether the reduction of binding and reactivity were due to the sequence variation or to the difference in the quaternary structure of the protein in unclear. The display of VAR2CSA epitopes targeted by functional antibodies might be modified on these extended proteins. If so, PM vaccine-induced antibodies against FCR3 and 3D7 alleles might have limited activity against isolates expressing extended VAR2CSA proteins. Investigation of *var2csa* structural diversity in isolates whose binding is not blocked by vaccine-induced antibodies may provide additional data to design VAR2CSA-based vaccines with broadly neutralizing activity.

In summary, we provide evidence of *var2csa* sequences with seven and eight DBL domains expressed by field isolates and demonstrate the importance of de novo sequence analysis to assess genetic diversity of *var2csa* in field isolates. Overall, *var2csa* sequences from approximatively 5% of isolates analyzed in this work exhibited DBL domain structures other than NTS-DBL1X-6ɛ, including extra DBL domains and a truncated *var2csa*, which will result in a nonfunctional protein if expressed. This study highlights the importance of ectopic recombination of DBL domains in *var2csa;* further investigations are required to understand the impact of these extended VAR2CSA in PM pathology.

## Methods

### *P. falciparum* isolates and RNA sequencing

Maternal (M200101) and child (C216085) isolates were obtained from participants in the Immuno-epidemiology (IMEP) study conducted in Ouélessébougou (Mali). A detailed description of the IMEP study has been previously reported^25^. Briefly, pregnant women were enrolled between November 2010 and October 2013 into a longitudinal cohort study of mother-infant pairs conducted in Ouélessébougou, Mali. The study site is located 80 km south of Bamako, an area of intense seasonal malaria transmission during the rainy season from July to December. Pregnant women aged 15–45 years without clinical evidence of chronic or debilitating illness were asked to participate in the study and gave signed informed consent after receiving a study explanation form and oral explanation from a study clinician in their native language. The protocol and study procedures were approved by the institutional review board of the National Institute of Allergy and Infectious Diseases at the US National Institutes of Health (ClinicalTrials.gov ID NCT01168271), and the Ethics Committee of the Faculty of Medicine, Pharmacy and Dentistry at the University of Bamako, Mali.

Binding phenotypes of each isolate were characterized over a 9-week period during in vitro culture with and without weekly synchronization of parasite developmental stage. Cultures were sampled weekly and stored in Trizol at –80 °C, and the level of parasite binding to CSA and CD36 was assessed as described elsewhere^26^. CSA-panned NF54 isolate (NF54_CSA_) was maintained in culture and similarly sampled and processed bi-weekly.

Total RNA was extracted from the Trizol-stored samples using RNeasy mini total RNA isolation kit and manufacturer’s protocol (Qiagen, USA). For next generation sequencing (NGS) analysis, a subset of samples corresponding to timepoints with differential CD36/CSA-binding levels was selected. Total RNA sample quality was assessed by a BioAnalyzer and an RNA integrity number (RIN) value ≥ 7 was obtained for all the samples. RNA sequencing was performed at the National Institutes of Health Intramural Sequencing Center (NISC). Briefly, sequencing libraries were constructed from ~1 µg total RNA using TruSeq® Stranded Total RNA Library Prep Globin (Illumina, Inc.) used according to manufacturer’s instructions. Adapters were Illumina 6-base single indexed. Amplification was performed using ten cycles, which was optimized for the input amount and to minimize the chance of over-amplification. Libraries were pooled in equimolar amounts for sequencing. The pooled libraries were sequenced on a HiSeq 4000 to achieve a minimum of 32 million 75 base read pairs. The data were processed using RTA version 2.7.7 and CASAVA 1.8.2. Raw FASTQ reads data were processed using in-house R package DuffyNGS, (https://github.com/robertdouglasmorrison/DuffyNGS), as originally described^27,28^.

### Extraction of DNA-seq and RNA-seq data from public databases

FASTQ datasets corresponding to both DNA-seq and RNA-seq from Mali, Gambia, New Guinea, Tanzania, and other lab strains (Study number and links are provided as Supplementary Data 3) were extracted from public databases. These FASTQ datasets and those generated in house were analyzed in this study.

### VAR2CSA sequence assembling and analysis

**Fig. 6:**
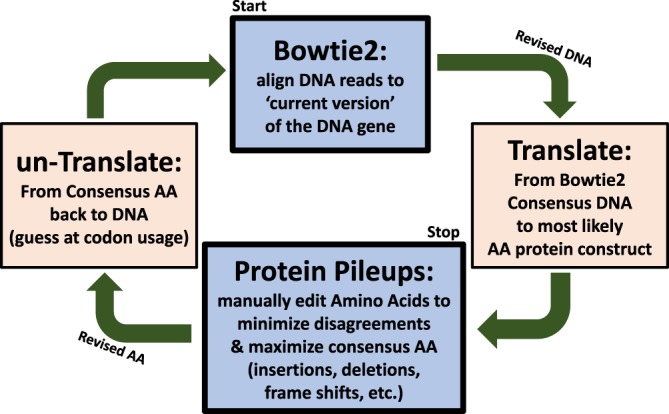
Flow chart of the consensus protein pileups methodology: an iterative process in two parallel worlds. Bowtie2 aligned raw reads to a reference genome were translated to amino acids (AA) sequences as peptides reads, manually edited in the consensus protein pileups tool, and untranslated back to DNA reads for re-alignment in Bowtie2. This cyclic process continues until convergence.

*Var2csa* transcripts were detected in 0/8, 5/6, and 3/3 of the selected samples from the child, maternal and NF54_CSA_ isolates, respectively, when using a transcription threshold of RPKM (Reads Per exon Kilobases per Million) > 5 (Supplementary Fig. 5). *Var2csa* raw sequencing reads from the maternal and NF54_CSA_ isolates were analyzed using the CPP tool (part of the publicly available R package DuffyNGS) through the analysis approach summarized in Fig. 6. Briefly, the DNA reads were first aligned to the 3D7 reference genome using Bowtie2^29^ to analyze the gene expression level for all genes in the genome. All raw reads that did not align to the 3D7 reference genome, referred to as no-hit reads, were kept for further analysis. All *var2csa* aligned and all no-hit raw DNA reads were converted into amino acid (AA) protein fragments (in all six reading frames), and all fragments with no internal stop codons were kept. Using the Bowtie2 consensus DNA sequence for *var2csa* as the initial version of the true *var2csa* DNA sequence, the best translation to the initial version of the protein sequence for the sample was generated. This initial protein version and the set of converted amino acid protein fragments were all used as inputs to the main CPP iterative process. Using R function pairwiseAlignment from the Biostrings package^30^ (available at BioConductor, www.bioconductor.org), each protein fragment was scored against the current protein version, and all high-scoring protein fragments were placed as protein pileups at their best scoring location on the current protein version. Each fragment’s alignment may contain some amino acid mismatches and/or gaps. After all fragments were aligned (or rejected for too low score) the most frequently observed fragment amino acid at each location along the protein was chosen as the new consensus amino acid at that location, indicating how the consensus pileups confirm or contradict the current protein version. Visual inspection of the pileups then allowed for manual editing of the current version of the protein (most frequently insertions, deletions and frame shifts) to better match the full-length protein to the pileups. Finally, the protein was reverse-translated back to DNA and the Bowtie alignment step was repeated against this newer reference, generating a new updated set of aligned raw reads to be translated into new protein fragments. The iterative process was repeated until the full-length protein converged to exactly match the consensus protein pileups. Results of the CPP analysis yielded AA and DNA sequences of *var2csa* that best represent the raw Illumina reads collected for that sample. Each maternal and NF54_CSA_ isolate was independently run through CPP to give DNA and AA sequences for *var2csa*.

### VAR2CSA sequence validation

In a second independent analysis approach, the raw DNA reads were submitted to Velvet^31^ and large DNA contigs were obtained. The DNA contigs were then translated in all six frames, and the one best-translated protein (DuffyNGS function DNAtoBestPeptide) was retained, followed by similarity analysis with *var2csa* sequence of 3D7, to identify the most likely DNA and AA sequence for *var2csa*. In a third analysis approach, a combination of Abyss and Trinity de novo assemblers as described elsewhere^32^ was used to generate the full-length (exon 1 and exon 2) VAR2CSA sequence of M200101.

### DBL domain similarity by the Edit distance in VAR2CSA

Similarity of the different domains of the *var2csa* sequence from the isolates to other reference sequences was analyzed using the R function pairwiseAlignment from the Biostrings package^30^, with the library of reference domain constructs from the VarDom 1.0 server. Edit distance (Edit_Dist) values were calculated by R function adist() and defined as the number of mismatched residues of AA in the domain to the reference domain sequence.

### Three-dimensional structure of the extra DBL domain in VAR2CSA_M200101_

The amino acid sequence corresponding to the extra DBL domain of *var2csa* in M200101 was submitted to the VarDom 1.0 server^16^ and I-TASSER^33^ for in silico prediction analysis using the default settings. The predicted model with a higher *C*-score (defined as a confidence score for estimating the quality of predicted models by I-TASSER^33^) was chosen.

### Amplification of the extra DBL domain in VAR2CSA_M200101_

Genomic DNA was extracted from the isolates using QIAamp DNA Blood Kit (Qiagen) according to the manufacturer’s instructions. Two pairs of primers (Supplementary Table 2) were designed to amplify fragments of sequence spanning DBL6ɛ and DBL7ɛ in VAR2CSA sequences from M200101 at two different timepoints, and two lab strains (NF54_CSA_ and FCR3_CSA_) were used as controls. PCR products were sequenced for sequence confirmation.

### Production of VAR2CSA recombinant proteins

Recombinant full-length M200101 DBL1X-7ɛ and NF54 DBL1X-6ɛ, as well as individual domain DBL7ɛ_M200101_ VAR2CSA proteins were expressed in Expi293 cells (ThermoFisher) as secreted and His-tagged proteins. One week after transfection, cell culture medium was loaded onto a HisTrap Excel NTA column. Eluted proteins were verified by sodium dodecyl sulfate–polyacrylamide gel electrophoresis (SDS-PAGE; Fig. 4a and Supplementary Fig. 6). Recombinant full-length M200101 DBL1X-7ɛ and NF54 DBL1X-6ɛ proteins were concentrated and further purified using a 100 kDa cutoff centrifugal filter unit. Both the concentrated full-length proteins and the individual DBL7ɛ_M200101_ were dialyzed into phosphate buffered saline (PBS) and stored at -80 °C until use.

### Binding of VAR2CSA recombinants to CSA

The ELISA binding assay on CSPG (Decorin) was performed as previously described^34^ with modifications. Briefly, 100 μL of CSPG at 5 μg per milliliter was coated on 96-wells plates, incubated overnight at 4 °C and blocked with PBS 1% BSA for 2 h at 37 °C with shaking. After discarding the blocking solution, serially diluted VAR2CSA recombinants from 10 μg per milliliter to 0.156 μg per milliliter were added to duplicated wells and incubated for 2 h at 37 °C with shaking, followed by three times washing and anti-His HRP-conjugated antibody (diluted 1/3000) incubation for 1 h at 37 °C with shaking, then washed. One-hundred microliters of 3,3′,5,5′-Tetramethylbenzidine (TMB) (SeraCare) was added to each well, then stopped with equal volume of stop solution (SeraCare) after 20 min and optical density (OD) was read at 450 nm. For this assay, MSP1 was used as a non-CSA-binding control antigen.

### ELISA and surface-labeling of VAR2CSA

Plasma pooled from malaria-exposed pregnant women (multigravidae and primigravidae) and children were assessed by ELISA to investigate the level of antibody binding to the full-length VAR2CSA_M200101_ with alternate domain structure and DBL7ɛ_M200101_ in comparison to full-length VAR2CSA_NF54_ and DBL5ɛ_FCR3_. Briefly, the antigens were coated at 1 μg per milliliter overnight at 4 °C and plates were blocked before adding plasma pools diluted at 5 × 10^−2^, 5 × 10^−3^, and 5 × 10^−4^ in duplicated wells then washed. HRP-conjugated anti-human IgG antibody (diluted 1/3000) was added for 1 h at room temperature. After a final wash, TMB (SeraCare) was added, then stopped with equal volume of stop solution (SeraCare) after 10 min and OD was read at 450 nm.

The surface expression of VAR2CSA by M200101 has been assessed in Flow cytometry using the anti-VAR2CSA-specific monoclonal PAM1.4 as previously described^35^. Briefly, M200101 IE at the trophozoite stage were incubated with PAM1.4 or a negative control monoclonal (MPE8) and washed. IE were then labeled with 0.1% SYBR green while bound antibodies were stained with PE-conjugated anti-human IgG. VAR2CSA-expressing cells were quantified using an LSRII flow cytometer (BD Biosciences, San Jose, CA) and analyzed with FlowJo, version 10, software (Tree Star, Inc.). The median fluorescence intensity (MFI) was determined and the background intensity from a well with no sample was subtracted from the detected MFI value for each sample.

### Statistics and reproducibility

Data were analyzed in GraphPad Prism 8 software (GraphPad Software, La Jolla, CA) to generate the graphs and statistical analysis. Plotted values were generated from three independent experiments and data were presented as mean ± SD. Difference in the isolates binding to the receptors and the naturally acquired antibodies binding to VAR2CSA antigens was evaluated with a Tukey’s multiple comparisons test following two-way ANOVA.

Statistical significance was defined as *p* < 0.05 and designated in figures as *p* ≤ 0.05 (*), *p* ≤ 0.01 (**), *p* ≤ 0.005 (***), and *p* ≤ 0.001 (****).

### Reporting summary

Further information on research design is available in the Nature Research Reporting Summary linked to this article.

## Supplementary information

Supplementary Information

Description of Additional Supplementary Files

Supplementary Data 1

Supplementary Data 2

Supplementary Data 3

Supplementary Data 4

Supplementary Data 5

Reporting Summary

## Data Availability

The raw FASTQ sequences of M200101 (SRR8176247) presented in this article have been submitted to the Gene Expression Omnibus (www.ncbi.nlm.nih.gov/geo) under project # PRJNA504638. The full-length DNA and amino acids sequences of VAR2CSA from isolates M200101, Mali_PS103, Mali_PS122, and C0111a0 are shown in Supplementary Data 4 and deposited to GenBank (accession number MN631060-MN631063). The source data plotted in figures are provided in Supplementary Data 5. All other data relevant to this study are available from the corresponding author on reasonable request.
