# Supplementary material for: Placental malaria vaccine candidate antigen VAR2CSA displays atypical domain architecture in some Plasmodium falciparum strains
Source: Commun Biol. 2019 Dec 6;2:457. doi: 10.1038/s42003-019-0704-z (PMC6897902; doi:10.1038/s42003-019-0704-z)
Supplement: Supplementary file 1 — Supplementary Information [file 42003_2019_704_MOESM1_ESM.pdf]

```
DBL6ε                                                                              DBL7ε
                           10        20        30        40        50        60        70        80        90       100
                   ....|....|....|....|....|....|....|....|....|....|....|....|....|....|....|....|....|....|....|....|
PILEUPS_DBL7e (467 aa)     PNVPETSSTKIDDIGSSKQNSFAIQHSKDKKLNKCALDENICKNYEDHKCNPKKNLDVLDEWNNLYLEDFQSKNKGVLIPPRRRQLCFTHMIKGPPRIQN
VELVEL_contig54 (467 aa)   PNVPETSSTKIDDIGSSKQNSFAIQHSKDKKLNKCALDENICKNYEDHKCNPKKNLDVLDEWNNLYLEDFQSKNKGVLIPPRRRQLCFTHMIKGPPRIQN
JOSE_contig1382 (467 aa)   PNVPETSSTKIDDIGSSKQNSFAIQHSKDKKLNKCALDENICKNYEDHKCNPKKNLDVLDEWNNLYLEDFQSKNKGVLIPPRRRQLCFTHMIKGPPRIQN

                                                             DBL7ε
                          110       120       130       140       150       160       170       180       190       200
                   ....|....|....|....|....|....|....|....|....|....|....|....|....|....|....|....|....|....|....|
PILEUPS_DBL7e (467 aa)     IDQFKNELLKGAVMEGKRLGEYYKNNSEKAIEAMTYSFADYADIIKGNDMIDTIPFKDIKRKLEQVLEQEEKSNNVLNTAEQWWKKNRKHLWNAMLCGYK
VELVEL_contig54 (467 aa)   IDQFKNELLKGAVMEGKRLGEYYKNNSEKAIEAMTYSFADYADIIKGNDMIDTIPFKDIKRKLEQVLEQEEKSNNVLNTAEQWWKKNRKHLWNAMLCGYK
JOSE_contig1382 (467 aa)   IDQFKNELLKGAVMEGKRLGEYYKNNSEKAIEAMTYSFADYADIIKGNDMIDTIPFKDIKRKLEQVLEQEEKSNNVLNTAEQWWKKNRKHLWNAMLCGYK

                                                             DBL7ε
                          210       220       230       240       250       260       270       280       290       300
                   ....|....|....|....|....|....|....|....|....|....|....|....|....|....|....|....|....|....|....|
PILEUPS_DBL7e (467 aa)     KTGYMPDAYVHLCIVPDTDETPQFLRWMIEWAKTFCNDKRNRGTSILKHCKDEIANNKNATNSSYKYECEKAAVNYVQWARKINEKWTGLSEKFKRSTSY
VELVEL_contig54 (467 aa)   KTGYMPDAYVHLCIVPDTDETPQFLRWMIEWAKTFCNDKRNRGTSILKHCKDEIANNKNATNSSYKYECEKAAVNYVQWARKINEKWTGLSEKFKRSTSY
JOSE_contig1382 (467 aa)   KTGYMPDAYVHLCIVPDTDETPQFLRWMIEWAKTFCNDKRNRGTSILKHCKDEIANNKNATNSSYKYECEKAAVNYVQWARKINEKWTGLSEKFKRSTSY

                                                             DBL7ε
                          310       320       330       340       350       360       370       380       390       400
                   ....|....|....|....|....|....|....|....|....|....|....|....|....|....|....|....|....|....|....|
PILEUPS_DBL7e (467 aa)     LPDAYKSYSPERYLTSKCGTCDCKYKDLKEIIDAYKEKTITDNFIDTIIDQAKNDNEQTPWSWLYPLSWPMWKIEAGIPKWTMKGDITIDWPDIKWPKID
VELVEL_contig54 (467 aa)   LPDAYKSYSPERYLTSKCGTCDCKYKDLKEIIDAYKEKTITDNFIDTIIDQAKNDNEQTPWSWLYPLSWPMWKIEAGIPKWTMKGDITIDWPDIKWPKID
JOSE_contig1382 (467 aa)   LPDAYKSYSPERYLTSKCGTCDCKYKDLKEIIDAYKEKTITDNFIDTIIDQAKNDNEQTPWSWLYPLSWPMWKIEAGIPKWTMKGDITIDWPDIKWPKID

                                                             DBL7ε
                          410       420       430       440       450       460
                   ....|....|....|....|....|....|....|....|....|....|....|....|....|..
PILEUPS_DBL7e (467 aa)     WEKPASKVRDSVHRISDIIFYIINNTHIIPTKDYKAHSDSSVTNQEIKVYKNLEERNLTPQLYERPE
VELVEL_contig54 (467 aa)   WEKPASKVRDSVHRISDIIFYIINNTHIIPTKDYKAHSDSSVTNQEIKVYKNLEERNLTPQLYERPE
JOSE_contig1382 (467 aa)   WEKPASKVRDSVHRISDIIFYIINNTHIIPTKDYKAHSDSSVTNQEIKVYKNLEERNLTPQLYERPE
```

Supplemental Figure 1: Alignments of DBL7ε fragment of VAR2CSAM200101 by the consensus protein pileups, velvet and Jose et al. tools. Truncated VAR2CSAM200101 sequence encompassing the end of DBL6ε and the DBL7ε domain as determined by the consensus protein pileups tool (PILEUPS_DBL7e) is aligned with same fragments identified by Velvet (VELVEL_contig54) and Jose et al. (JOSE_contig1382) tools.

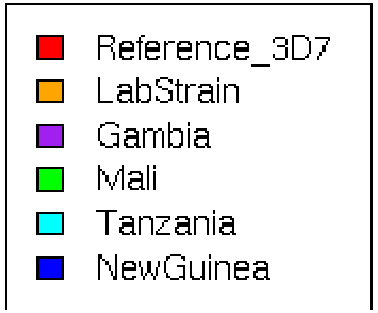

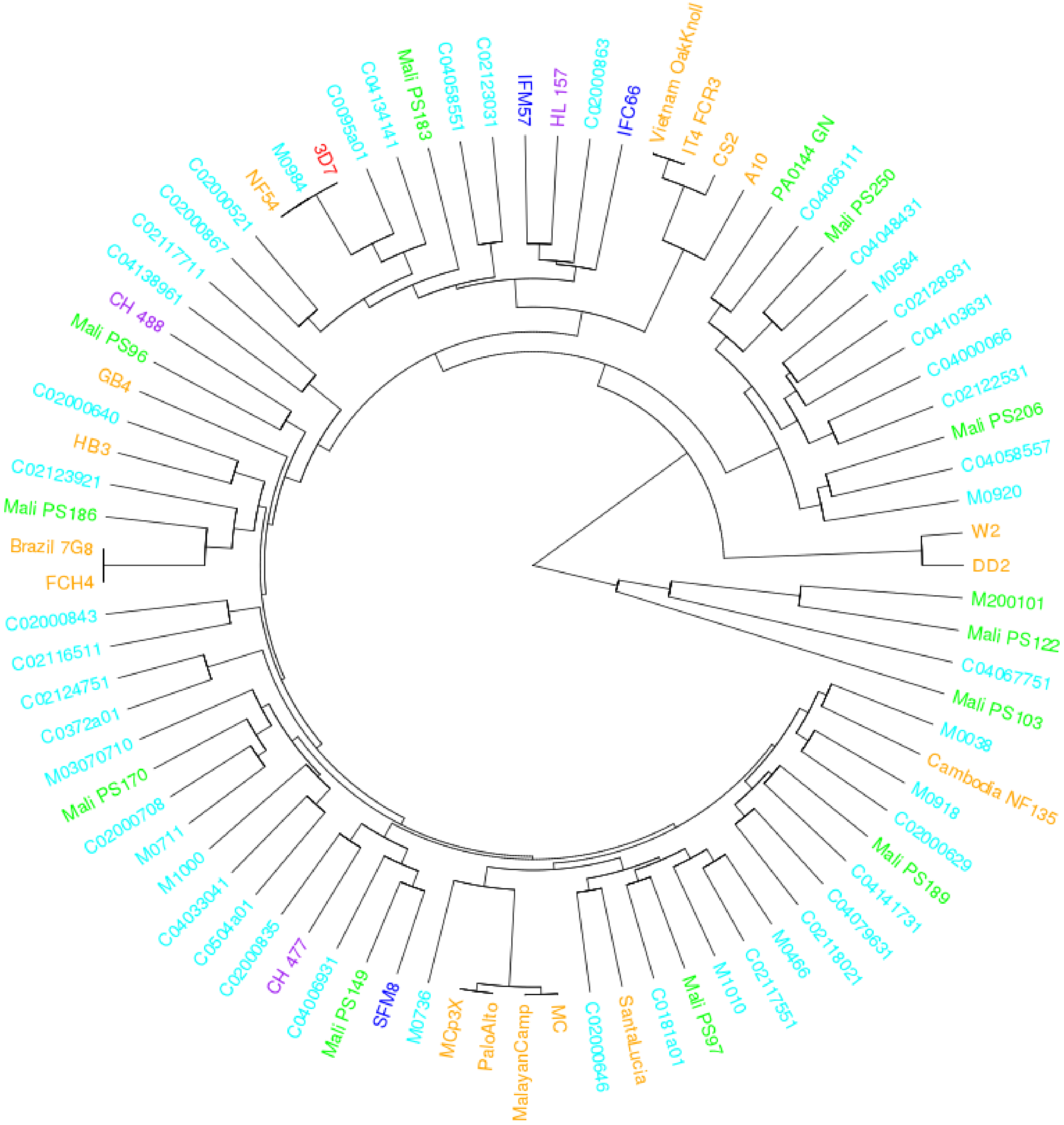

Supplementary Figure 2:
Phylogenetic tree showing of full-length *var2csa* sequences from lab strains and isolates from different geographic locations. The phylogenetic trees were made using the R package “ape”.[1]

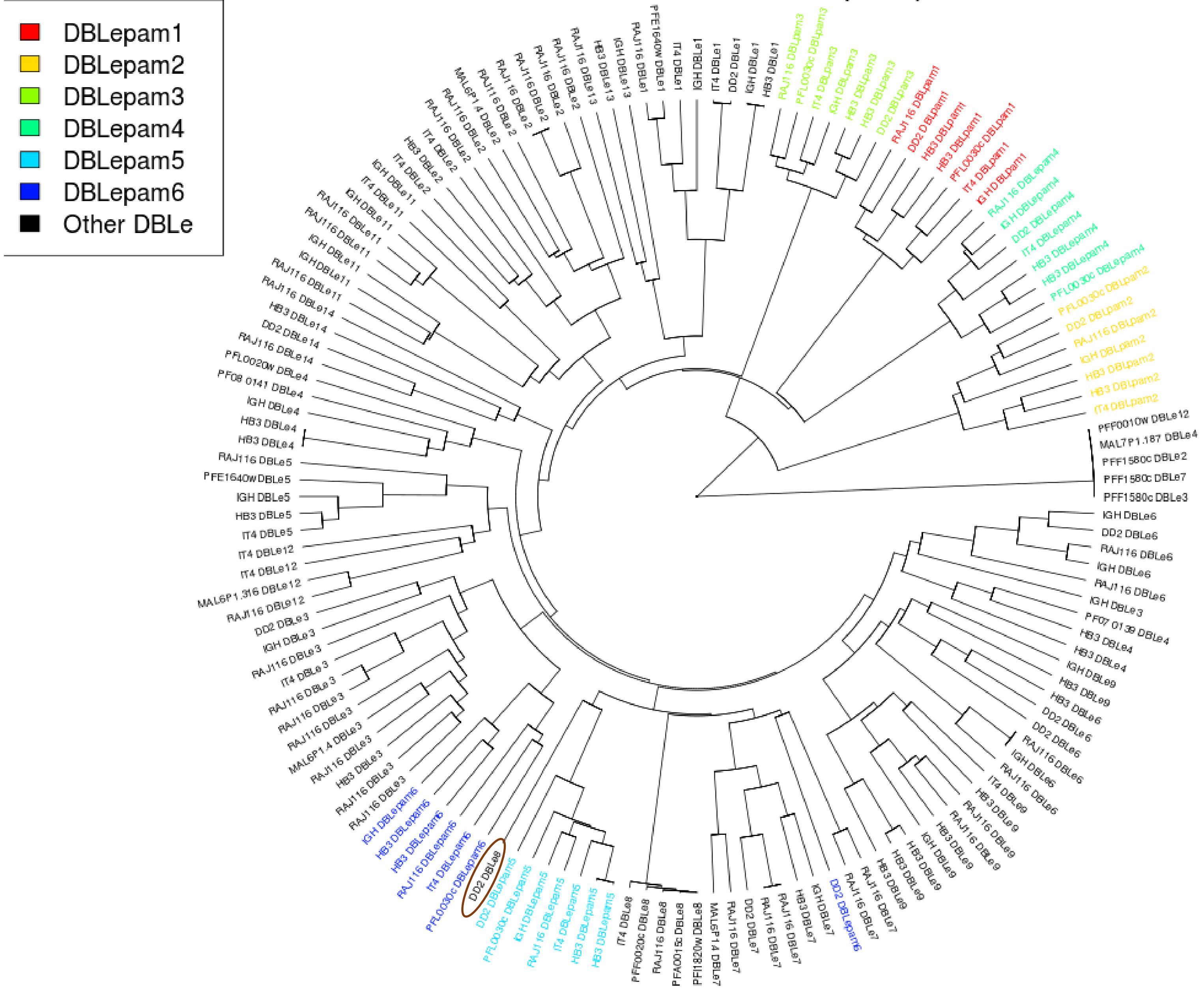

Supplementary Figure 3: Phylogenetic tree showing classification of DBLε sequences of *var* genes from VarDom 1.0 server. 148 sequences of DBLε of *var* genes from 3D7, HB3, DD2, IT4/FCR3, RAJ116 and IGH used VarDom 1.0 database. Clusters of the DBL domains corresponding to VAR2CSA are highlighted in different colors. The DBLε8 domain of *DD2var28* which was identified as DBL7ε in VAR2CSA sequences from M200101 and Mali_PS122 isolates, is indicated in red circle clustering with DBL5ε VAR2CSA domains. Trees were generated using the R package "ape".[1]

Supplementary Figure 4: Serial dilution of plasma antibody binding VAR2CSA recombinants. Plasma pools from Malian multigravidae, primigravidae and children were diluted at 1:500, 1:5000 and 1:50000 and characterized for binding to recombinants **(a)** DBL7ε$_{M200101}$; **(b)** full-length VAR2CSA$_{M200101}$ and **(c)** DBL5ε$_{FCR3}$ and **(d)** full-length VAR2CSA$_{NF54}$. O.D values from three independent experiments are reported.

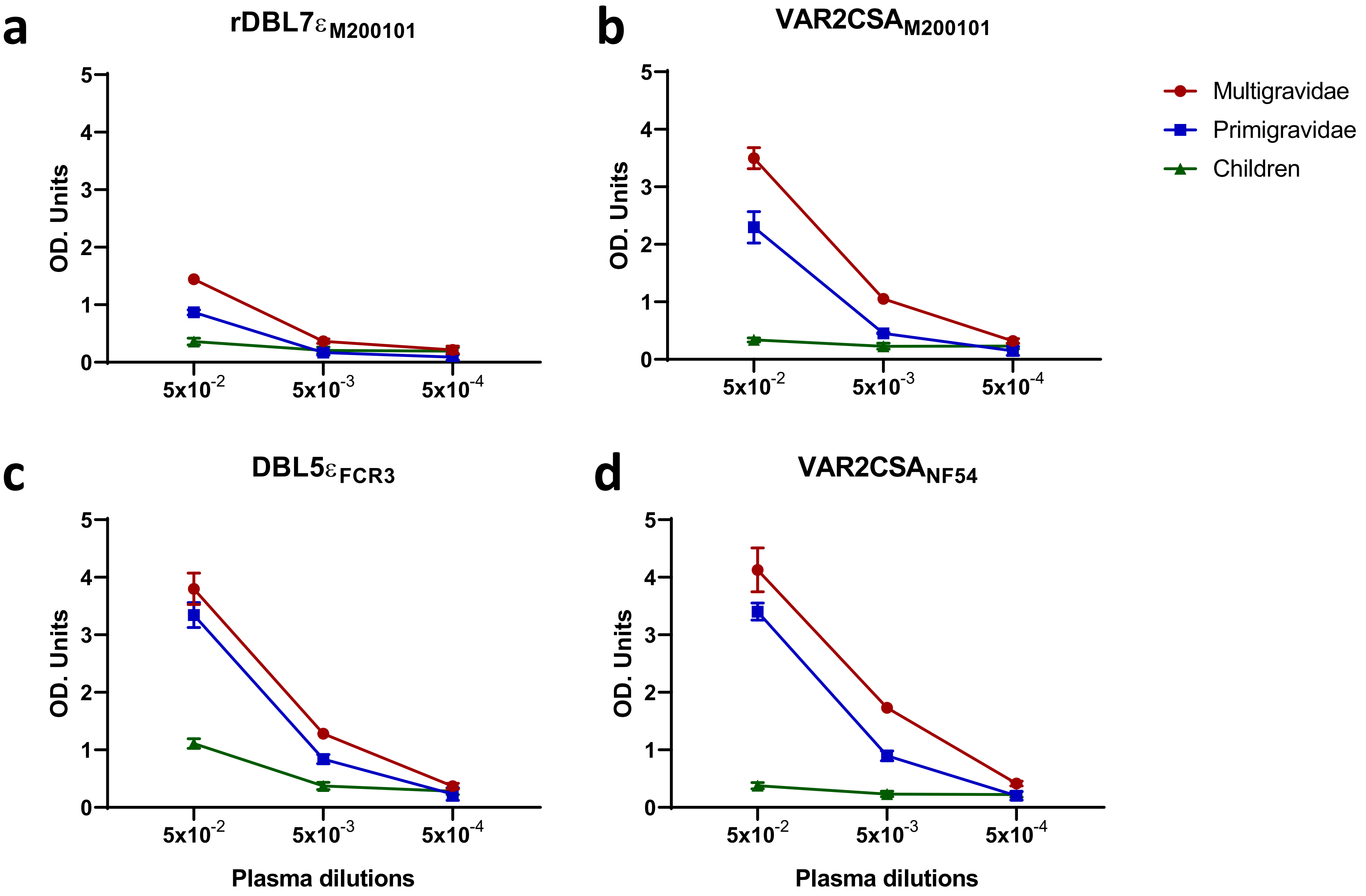

Supplemental Figure 5

Transcript abundance of var2csa in NF54, the child and maternal isolates. Transcriptomic data of var2csa expression by NF54 (in red), the maternal (in purple) and the child (in blue) isolates at different timepoints of the long-term culture were analyzed and presented as **(a)** RPKM and **(b)** Read count. Dashed lines indicate the threshold of detection and the minimum depth of coverage for assembly of var2csa by the CPP tool.

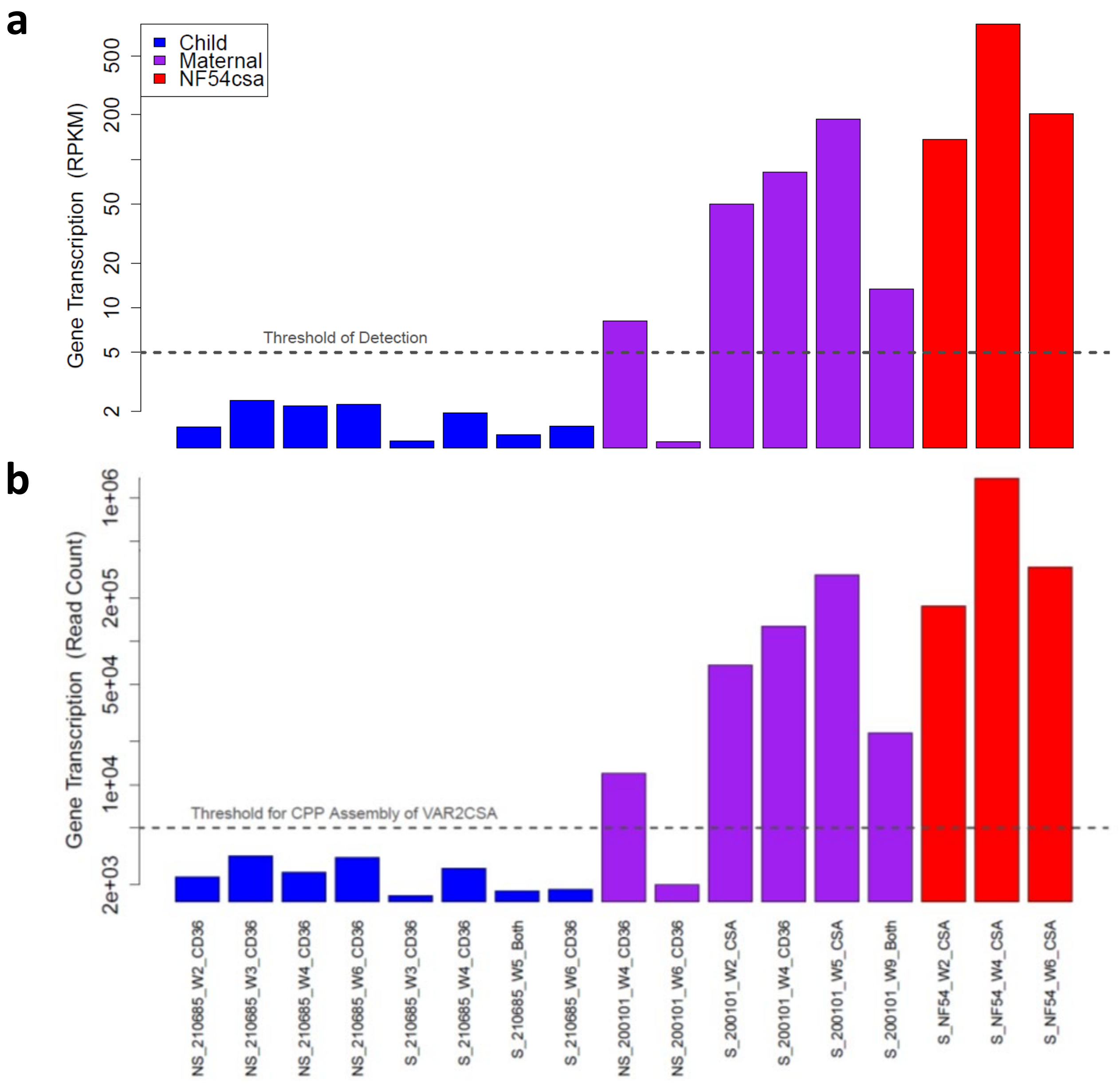

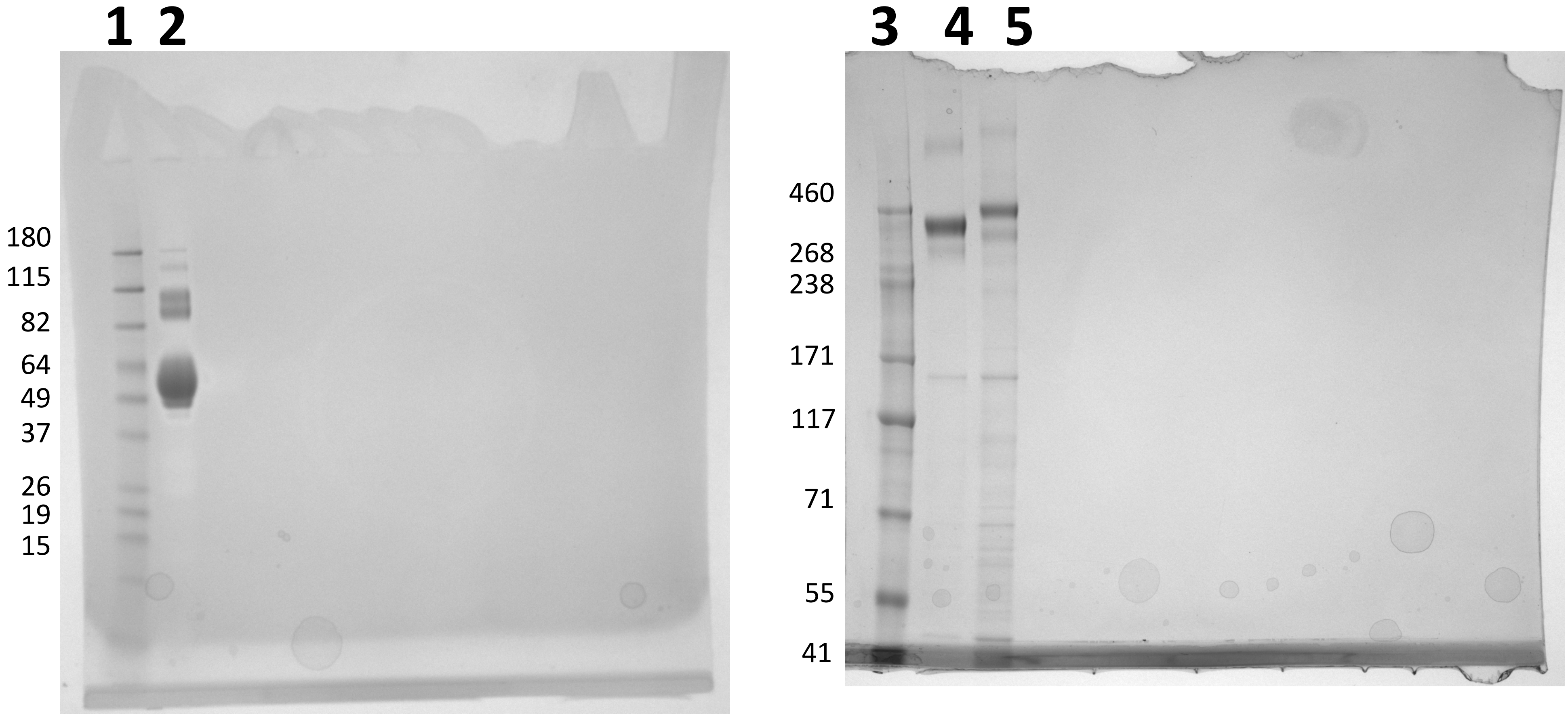

Supplementary Figure 6: SDS-PAGE analysis of recombinant proteins.

Uncropped images of SDS-PAGE analysis of recombinant proteins. Ten micrograms of purified DBL7εM200101 VAR2CSA (lane 2) was loaded on a 4-12% bis-tris gel and Coomassie stained. One microgram of purified full-length M200101 DBL1x-7ε (lane 5) and NF54 DBL1x-6ε (lane 4) were loaded on a 3-8% tris-acetate gel and Coomassie stained. Lane 1 and 3 are molecular weight markers (kDa).

Rank	PDB Hit	Domain name*	TM-score	RMSD	Identity	Coverage
1	2yk0A	NTS-DBL1α[2]	0.957	1.18	0.172	0.977
2	2xu0A	NTS-DBL1α[3]	0.763	1.85	0.195	0.789
3	5mzaA	DBLβ of PF11_0521[4]	0.648	3.08	0.206	0.73
4	4gf2A	PfEBA-140/BAEBL[5]	0.593	3.95	0.153	0.702
5	1zroB	EBA-175[6]	0.57	3.76	0.177	0.664
6	3bqkA	DBL3X VAR2CSA[7]	0.549	3.96	0.159	0.654
7	4p1tA	DBL3X-DBL4ε[8]	0.547	4.53	0.127	0.679
8	2y8dA	DBL6ε VAR2CSA[9]	0.543	3.17	0.251	0.614
9	3rrcA	PvDBP[10]	0.528	3.66	0.205	0.611
10	2wauA	DBL6ε VAR2CSA[11]	0.52	3.43	0.242	0.6

Supplementary Table 1: Top 10 identified structural analogs of DBL7ε $VAR2CSA_{M200101}$ in PDB. Ranking of proteins is based on TM-score of the structural alignment between the query structure and known structures in the PDB library. TM-score has a value between 0 to 1, where 1 indicates a perfect match between two structures. RMSD is the root-mean-square deviation between residues that are structurally aligned by TM-align. The percentage sequence identity in the structurally aligned region is indicated. The reported coverage of the alignment by I-TASSER is equal to the number of structurally aligned residues divided by length of the query protein.

* Reference is indicated for each domain structure

Supplemental Table 2 List of primers for the amplification of DBL6ε-DBL7ε fragments of VAR2CSA$_{M200101}$

	Sequence (5'->3')
**F1_DBL6e**	TGATGAAAAGCCCGACATCTA
**R1_TMD**	ACTGCACCTATGGTAGGAACG
**F2_DBL7e**	GTGCTGATTCCTCCAAGACG
**R2_TMD**	CCGCTACTGCACCTATGGTA

Supplementary references

1. Paradis, E. & Schliep, K. ape 5.0: an environment for modern phylogenetics and evolutionary analyses in R. *Bioinformatics* **35**, 526-528 (2018).
2. Vigan-Womas, I. *et al.* Structural basis for the ABO blood-group dependence of Plasmodium falciparum rosetting. *PLoS Pathog.* **8**, e1002781 (2012).
3. Juillerat, A. *et al.* Structure of a Plasmodium falciparum PfEMP1 rosetting domain reveals a role for the N-terminal segment in heparin-mediated rosette inhibition. *Proc. Natl. Acad. Sci. U. S. A.* **108**, 5243–5248 (2011).
4. Lennartz, F. *et al.* Structure-Guided Identification of a Family of Dual Receptor-Binding PfEMP1 that Is Associated with Cerebral Malaria. *Cell Host Microbe* **21**, 403–414 (2017).
5. Lin, D. H., Malpede, B. M., Batchelor, J. D. & Tolia, N. H. Crystal and solution structures of Plasmodium falciparum erythrocyte-binding antigen 140 reveal determinants of receptor specificity during erythrocyte invasion. *J. Biol. Chem.* **287**, 36830–36836 (2012).
6. Tolia, N. H., Enemark, E. J., Sim, B. K. L. & Joshua-Tor, L. Structural basis for the EBA-175 erythrocyte invasion pathway of the malaria parasite Plasmodium falciparum. *Cell* **122**, 183–193 (2005).
7. Higgins, M. K. The structure of a chondroitin sulfate-binding domain important in placental malaria. *J. Biol. Chem.* **283**, 21842–21846 (2008).
8. Gangnard, S. *et al.* Structure of the DBL3X-DBL4ε region of the VAR2CSA placental malaria vaccine candidate: insight into DBL domain interactions. *Sci. Rep.* **5**, 14868 (2015).
9. Gangnard, S. *et al.* Structural and immunological correlations between the variable blocks of the VAR2CSA domain DBL6ε from two Plasmodium falciparum parasite lines. *J. Mol. Biol.* **425**, 1697–1711 (2013).
10. Batchelor, J. D., Zahm, J. A. & Tolia, N. H. Dimerization of Plasmodium vivax DBP is induced upon receptor binding and drives recognition of DARC. *Nat. Struct. Mol. Biol.* **18**, 908–914 (2011).
11. Khunrae, P., Philip, J. M. D., Bull, D. R. & Higgins, M. K. Structural comparison of two CSPG-binding DBL domains from the VAR2CSA protein important in malaria during pregnancy. *J. Mol. Biol.* **393**, 202–213 (2009).
