# Supplementary material for: Placental malaria vaccine candidate antigen VAR2CSA displays atypical domain architecture in some Plasmodium falciparum strains
Source: Commun Biol. 2019 Dec 6;2:457. doi: 10.1038/s42003-019-0704-z (PMC6897902; doi:10.1038/s42003-019-0704-z)
Supplement: Supplementary file 2 — Description of Additional Supplementary Files [file 42003_2019_704_MOESM2_ESM.docx]

**Description of additional supplementary items**

**Supplementary Data 1:** Sanger sequencing result of the PCR products from the amplification of DBL6ɛ-7ɛ VAR2CSA_M200101_

**Supplementary Data 2:** Domain details of the *var2csa* sequences analyzed by the CPP tool

**Supplementary Data 3:** FastQ links of analyzed samples for DBL7ɛ screening.

**Supplementary Data 4:** DNA and protein sequences of full-length VAR2CSA_M200101_

**Supplementary Data 5:** Source data underlying the graphs
