# Supplementary material for: Placental malaria vaccine candidate antigen VAR2CSA displays atypical domain architecture in some Plasmodium falciparum strains
Source: Commun Biol. 2019 Dec 6;2:457. doi: 10.1038/s42003-019-0704-z (PMC6897902; doi:10.1038/s42003-019-0704-z)
Supplement: Supplementary file 3 — Supplementary Data 1 [file 42003_2019_704_MOESM3_ESM.docx]

**Supplementary data 1:** Sanger sequencing result of the PCR products from the amplification of DBL6ɛ-7ɛ VAR2CSAM200101

>M200101_F1

NNNNNTNNNNNNNCGGAATATAGTGGAATTGATAGTGAAACTTGGTGGGACTCGAATAAATATCATGTATGGGAATCCATGTTATGTGGATACAAAAAAGCCTACGGAAAATTTTCAGAAGATGTTGAAAAAATTCGTGAAATACCTAATAATGATAACGTAAATCAATTTTTACGGTGGTTTACAGAATGGGCACAAGATTTTTGTTATCACCAAGCGGAGGAAATTAAAAAATTAGAGGAAGAATGTAACTTTAATACATGTGAAGAAGCAAATGTTCGTCAAAAATCAGAATGTCAACATCAGTGTAATAAATATAAAAAATTTTTAAGAAAATGGAAAGCTCAATATAACAGACAAAATATTAAATATGAAGGATTAACAGACTCAATTAATATAATAAAAAATAAGGAAGCTCCTAAATTTTTGACGGAACATTGTAAAGAAGAATGCTCATGTTTTCAATCCACAAACGTCAATAATGTTATTAATATGTTTGAAAAATTACCTGATGAGTATATAAAAAAATGTCCTTGTCCAAATGTACCTGAAACATCTTCAACTAAAATAGATGATATCGGGAGTTCAAAACAGAATTCATTTGCAATCCAACATTCAAAGGATAAGAAATTGAATAAATGCGCATTGGATGAAAATATCTGCAAAAATTATGAAGATCATAAATGTAACCCCAAAAAAAACCTTGATGTTCTTGATGAATGGAATAATTTATATCTGGAAGATTTTCAATCTAAAAATAAAGGCGTGCTGATTCCTCCAAGACGAAGACAGTTATGCTTTACGCATATGATTAAAGGTCCTCCCAGAATACAAAATATCGATCAATTTAAGAATGAACTATTAAAAGGTGNGGTTATGGAAGGTAAACGTTTAGGAGAATATTATAAAAATAATAGTGAAAAAGCAATTGAAGCAATGACATACAGTTTTGCTGATTATGCAGATATAATTAAAGGAAATGATATGATAGATACCATACCATTCAAGGATATTAAGCGAAAATTAGAACAAGTTCTTGAACAANAAAAAANTCGNNAATGTTCTTANNNAGCANAACAATGGTGGAAAAAANNAAAAANNTTTGTGGAATGCGATNNTNNNNGGANNCAAAAAACAGGANNNTGCCTGATGCNACGTNNCCTTTGCATGTACCNGANNCGATGAANTCCNNATTCT

**EYSGIDSETWWDSNKYHVWESMLCGYKKAYGKFSEDVEKIREIPNNDNVNQFLRWFTEWAQDFCYHQAEEIKKLEEECNFNTCEEANVRQKSECQHQCNKYKKFLRKWKAQYNRQNIKYEGLTDSINIIKNKEAPKFLTEHCKEECSCFQSTNVNNVINMFEKLPDEYIKKCPCPNVPETSSTKIDDIGSSKQNSFAIQHSKDKKLNKCALDENICKNYEDHKCNPKKNLDVLDEWNNLYLEDFQSKNKGVLIPPRRRQLCFTHMIKGPPRIQNIDQFKNELLKGXVMEGKRLGEYYKNNSEKAIEAMTYSFADYADIIKGNDMIDTIPFKDIKRKLEQVLEQ**

>M200101_F2

NNNNNNNNNNCNNATGANNNNNNCCNNNNAGAATACAAAATATCGATCAATTTAAGAATGAACTATTAAAAGGTGCTGTTATGGAAGGTAAACGTTTAGGAGAATATTATAAAAATAATAGTGAAAAAGCAATTGAAGCAATGACATACAGTTTTGCTGATTATGCAGATATAATTAAAGGAAATGATATGATAGATACCATACCATTCAAGGATATTAAGCGAAAATTAGAACAAGTTCTTGAACAAGAAGAAAAATCGAACAATGTTCTTAACACAGCAGAACAATGGTGGAAAAAAAATAGAAAACATTTGTGGAATGCGATGTTATGTGGATACAAAAAAACAGGATACATGCCTGATGCATACGTACACCTTTGCATTGTACCCGATACCGATGAAACTCCTCAATTCTTACGATGGATGATAGAATGGGCTAAAACATTCTGTAATGACAAAAGAAATAGAGGAACGTCTATCTTGAAACATTGTAAGGATGAAATTGCTAACAATAAAAACGCTACAAACTCAAGTTATAAATATGAATGCGAAAAGGCTGCTGTGAATTATGTACAATGGGCTAGAAAAATAAATGAAAAATGGACTGGATTATCTGAAAAATTTAAAAGATCCACAAGTTATCTTCCTGATGCATATAAATCATATTCACCTGAACGATATTTAACATCAAAATGTGGTACCTGTGATTGTAAATATAAGGATTTAAAAGAAATAATTGATGCGTACAAGGAAAAAACAATAACAGATAATTTCATTGATACAATTATTGATCAAGCAAAAAACGATAACGAACAAACTCCTTGGTCATGGCTATATCCTTTATCGTGGCCTATGTGGAAAATAGAAGCTGGAATTCCTAAATGGACCATGAAAGGGAGATATAACCAATTGATTGGCCAGACATTAAATGGCCTAAAATTGANTGGGAAAAACCTGCCTCAAAAGTAAAGAGATTCAGTTCACCGAANNTCNNANNNTTATTTTTTTNNNTAATAAACAATACNCATATAATACCCACTAAANATTATAAAGCACATTCCGATTCTTCTGTTACAATCAGGAAATAAAAGTGTATAAAAATTTAGAAGAACGTAACCTAACTCCCCATTNNNGAACGTCCNAATNNTCCNCNCCCNNCAGGGGGGGNNNNANAAANNNNTNAGNA

**RIQNIDQFKNELLKGAVMEGKRLGEYYKNNSEKAIEAMTYSFADYADIIKGNDMIDTIPFKDIKRKLEQVLEQEEKSNNVLNTAEQWWKKNRKHLWNAMLCGYKKTGYMPDAYVHLCIVPDTDETPQFLRWMIEWAKTFCNDKRNRGTSILKHCKDEIANNKNATNSSYKYECEKAAVNYVQWARKINEKWTGLSEKFKRSTSYLPDAYKSYSPERYLTSKCGTCDCKYKDLKEIIDAYKEKTITDNFIDTIIDQAKNDNEQTPWSWLYPLSWPMWKIEAGIPKWTMKG**

>M200101_R1

NNNNNNNNGNANGTATCCTGTTTTTTTGTATCCACATAACATCGCATTCCACAAATGTTTTCTATTTTTTTTCCACCATTGTTCTGCTGTGTTAAGAACATTGTTCGATTTTTCTTCTTGTTCAAGAACTTGTTCTAATTTTCGCTTAATATCCTTGAATGGTATGGTATCTATCATATCATTTCCTTTAATTATATCTGCATAATCAGCAAAACTGTATGTCATTGCTTCAATTGCTTTTTCACTATTATTTTTATAATATTCTCCTAAACGTTTACCTTCCATAACAGCACCTTTTAATAGTTCATTCTTAAATTGATCGATATTTTGTATTCTGGGAGGACCTTTAATCATATGCGTAAAGCATAACTGTCTTCGTCTTGGAGGAATCAGCACGCCTTTATTTTTAGATTGAAAATCTTCCAGATATAAATTATTCCATTCATCAAGAACATCAAGGTTTTTTTTGGGGTTACATTTATGATCTTCATAATTTTTGCAGATATTTTCATCCAATGCGCATTTATTCAATTTCTTATCCTTTGAATGTTGGATTGCAAATGAATTCTGTTTTGAACTCCCGATATCATCTATTTTAGTTGAAGATGTTTCAGGTACATTTGGACAAGGACATTTTTTTATATACTCATCAGGTAATTTTTCAAACATATTAATAACATTATTGACGTTTGTGGATTGAAAACATGAGCATTCTTCTTTACAATGTTCCGTCAAAAATTTAGGAGCTTCCTTATTTTTTATTATATTAATTGAGTCTGTTAATCCTTCATATTTAATATTTTGTCTGTTATATTGAGCTTTCCATTTTCTTAAAAATTTTTTATATTTATTACACTGATGTTGACATTCTGATTTTTGACGAACATTTGCTTCTTCACATGTATTAAAGTTACATTCTTCCTCTAATTTTTTAATTTCCTCCGCTTGGTGATAACAAAAATCTTGTGCCCATTCTGTAAACCACCGTAAAAATTGATTTACGTTATCATTATTAGGTATTTCACGAATTTTTTCAACATCTTCTGAAAATTTTCCGTAGGCTTTTTTGTATCNNNATAACAGGGATTCNNNTACATGATATTTATTCNAGTCCCCCNAGTTTNNTATCANTTC

**YKNNSEKAIEAMTYSFADYADIIKGNDMIDTIPFKDIKRKLEQVLEQEEKSNNVLNTAEQWWKKNRKHLWNAMLCGYKKTGYMPDAYVHLCIVPDTDETPQFLRWMIEWAKTFCNDKRNRGTSILKHCKDEIANNKNATNSSYKYECEKAAVNYVQWARKINEKWTGLSEKFKRSTSYLPDAYKSYSPERYLTSKCGTCDCKYKDLKEIIDAYKEKTITDNFIDTIIDQAKNDNEQTPWSWLYPLSWPMWKIEAGIPKWTMKGDITIDWPDIKWPKIDWEKPASKVRDSVHRISDIIFYIINNTHIIPTKDYKAHSDSSVTNQEIKVYKNLEERNLTP**

>M200101_R2

NNNNNNNNNNNNNNNNNNNNNNTTTNGGGNNNNNNNNNTACNNNNNNTNNNNNAATTTTTATACACTTTTATTTTCCTGATTTGTAACAGAAGAATCGGAATGTGCTTTATAATCTTTAGTGGGTATTATATGTGTATTGTTTATTATATAAAAAATAATATCTGATATTCGGTGAACTGAATCTCTTACTTTTGAGGCAGGTTTTTCCCAGTCAATTTTAGGCCATTTAATGTCTGGCCAATCAATTGTTATATCTCCCTTCATGGTCCATTTAGGAATTCCAGCTTCTATTTTCCACATAGGCCACGATAAAGGATATAGCCATGACCAAGGAGTTTGTTCGTTATCGTTTTTTGCTTGATCAATAATTGTATCAATGAAATTATCTGTTATTGTTTTTTCCTTGTACGCATCAATTATTTCTTTTAAATCCTTATATTTACAATCACAGGTACCACATTTTGATGTTAAATATCGTTCAGGTGAATATGATTTATATGCATCAGGAAGATAACTTGTGGATCTTTTAAATTTTTCAGATAATCCAGTCCATTTTTCATTTATTTTTCTAGCCCATTGTACATAATTCACAGCAGCCTTTTCGCATTCATATTTATAACTTGAGTTTGTAGCGTTTTTATTGTTAGCAATTTCATCCTTACAATGTTTCAAGATAGACGTTCCTCTATTTCTTTTGTCATTACAGAATGTTTTAGCCCATTCTATCATCCATCGTAAGAATTGAGGAGTTTCATCGGTATCGGGTACAATGCAAAGGTGTACGTATGCATCAGGCATGTATCCTGTTTTTTTGTATCCACATAACATCGCANTCCACAAATGTTTTCTATTTTTTTTCCACCATTGTTCTGCTGTGTTAAGAACATTGTTCGATTTTTCTTCTTGTTCAAGAACTTGTTCTAATTGTCGCTTAATATCCTTGAATGGTATGGTATCTATCAAATCATTTCCTTTAATNATATCTGCATAATCAGCAAAACTGTANGTCATTGCTTCAATTGCNNTTTCACTATAATTNTNNAATNTNCTCCTAACNTTTACCTTCCNAACANNACCTTTTAATANTTCATCNNAAATTGANNNNNNNTTGTATTCTGGAAGANNNNAANNNNNNCAAANNGAAAACTGNNNNNNA

**AMLCGYKKTGYMPDAYVHLCIVPDTDETPQFLRWMIEWAKTFCNDKRNRGTSILKHCKDEIANNKNATNSSYKYECEKAAVNYVQWARKINEKWTGLSEKFKRSTSYLPDAYKSYSPERYLTSKCGTCDCKYKDLKEIIDAYKEKTITDNFIDTIIDQAKNDNEQTPWSWLYPLSWPMWKIEAGIPKWTMKGDITIDWPDIKWPKIDWEKPASKVRDSVHRISDIIFYIINNTHIIPTKDYKAHSDSSVTNQE**

>AmplifiedPCRSeqDBL6-7_M200101_protein

**EYSGIDSETWWDSNKYHVWESMLCGYKKAYGKFSEDVEKIREIPNNDNVNQFLRWFTEWAQDFCYHQAEEIKKLEEECNFNTCEEANVRQKSECQHQCNKYKKFLRKWKAQYNRQNIKYEGLTDSINIIKNKEAPKFLTEHCKEECSCFQSTNVNNVINMFEKLPDEYIKKCPCPNVPETSSTKIDDIGSSKQNSFAIQHSKDKKLNKCALDENICKNYEDHKCNPKKNLDVLDEWNNLYLEDFQSKNKGVLIPPRRRQLCFTHMIKGPPRIQNIDQFKNELLKGAVMEGKRLGEYYKNNSEKAIEAMTYSFADYADIIKGNDMIDTIPFKDIKRKLEQVLEQEEKSNNVLNTAEQWWKKNRKHLWNAMLCGYKKTGYMPDAYVHLCIVPDTDETPQFLRWMIEWAKTFCNDKRNRGTSILKHCKDEIANNKNATNSSYKYECEKAAVNYVQWARKINEKWTGLSEKFKRSTSYLPDAYKSYSPERYLTSKCGTCDCKYKDLKEIIDAYKEKTITDNFIDTIIDQAKNDNEQTPWSWLYPLSWPMWKIEAGIPKWTMKGDITIDWPDIKWPKIDWEKPASKVRDSVHRISDIIFYIINNTHIIPTKDYKAHSDSSVTNQEIKVYKNLEERNLTP**
