# Supplementary material for: Placental malaria vaccine candidate antigen VAR2CSA displays atypical domain architecture in some Plasmodium falciparum strains
Source: Commun Biol. 2019 Dec 6;2:457. doi: 10.1038/s42003-019-0704-z (PMC6897902; doi:10.1038/s42003-019-0704-z)
Supplement: Supplementary file 6 — Supplementary Data 4 [file 42003_2019_704_MOESM6_ESM.docx]

>M200101_VAR2CSA_exon1andexon2_DNA_seq

ATGGATAGTAAAACCACTATTGCTGAAAAAATTGAAGAATACTTAAAGGAAAAATCCAATGATTCTAAAATAGACCAATCGTTGAAAGCTGATCCTAGTGAAGTGCAGTACTATAGAAGTGGAGGTGATGGATATTACTTAAAAAATAATATTTGCAAAATTACTGTGAATCATTCAGATTCTGGAACAAATGATCCTTGTGATAGAAAAGAACTTTCTTATGGCGATAATGACCAATGGAAATGTGACGAAAATTTATCTAAAGTAAGTGGAAAACCTGAAAATATATGTGTTCCTCCGAGAAGACAACGTATGTGCATTAAGAATTTAGAAAAATTAAATGTTGATAAAATTAGGGATAAACATGCATTTTTGGCAGATGTATTACTTACGGCCAGAAATGAAGGAGAAAGAATTATACTATATCATCCAGATACAAATAGTTCTAATGTTTGTGTTGCGTTAGAAAGAAGTTTTGCTGATCTTGCAGATATTATTAGAGGTAGAGATGGGAACAAGTGTGAAACAAAATCCCCAAATAATGTTGAAGAACTAATAAAAAAATTCTTCGAAAAAAATTACAGATCAAATGAAGAATATAAAAGAAAATATCGAAATGATGATGAAAATTATAAGAAATTACGAGAAGCTTGGTGGAATGCTAATAGACAAAAGGTGTGGGAAGTTATTACCTGTAGTGCACGAAGTAACGATTTACTCATAAAACGTCGATGGACAACATCTAAGGAGTCTAATGGAGAAAATAAATTGGAATTGTTCCGCAAATGTGGCCATTATGAAAAAGAGGTTCCTACCAAATTAGATTATGTCCCTCAATTTTTAAGGTGGTTAACAGAATGGATAGCGGATTTATATAGAGAGAAGCAAAATCTGATCGATGACATGGAGAGGCACCGTGAAGAGTGTACAAGAGAGGATGATAAATCTAAAGAAGTTACATCATATTGTAATATGTGTAAAGACAAATGTAAGAAATATTGTGAATGTGTGAAGAAATGGAAGACCGAATGGGAAAATCAAAAAAATAAATATAATGATTTATATGAACAAGAAAACGAAACTTCGCAAAAAAATACATCAAGATATGATGATTATGTTAAAGAATTTTTTGAAAAACTTAATGAAGCTAATTATAAGTCTCTTGATGATTATATAAAGGGTGATCCTTATTTCGCAGAATATGCAACCAAATTATCATTTATTTTAAATTCATCAGATGCTAATAATCCGTCTGAAAAAATACAAAAAAATAATGATGAAGTATGTAACTGTAATGAATCAGGAATTTCATCTGTTGAACAGGCATCAATATCTGATCCGTCGTCGAATAAAACATGTAACACACATAGCTCTATAAAAACTAATAAGAAAAAAGAATGTAAACATGTAAAGTTGGGTGTTCGTGAAAATGATAAGGTTTTGAAAATATGCGTAATTGAGGACACTTCCTTAAGTGGTGTTGAAAATTGTTGTTTCAAAGATTTATTGGGAATTCTTCAAGAACCTCGAATTGATAAAAACCAAAGTGGATCTAGTTCTAATGGTAGTTGTGATAAAAATAGTGAGGAAATATGTCAAAAGAAATTAGATGAAGCCCTTGCATCTTTACATAATGGTTATAAATGCGACAAATGTAAATCTGGAACATCAACAGTTAACAAAAATTGGATATGGAAAAAATTCCCTGGTAATGGGGAAGGATTACAAAAAGAATATGCTAATACCATAGGTTTACCCCCAAGAACACATTCGTTATATTTAGGAAATCTACCTAAACTTGAAAATGTGTGCGAAGATGTAAAGGATATTAATTTTGATACAAAAGAGAAATTCCTAGCAGGATGCTTAATTGCTGCTTTCCATGAAGGAAAAAATTTAAAAACAACATACCCTCAAAATAAAAAAAAATTATGCAAAGCTTTAAAATACAGTTTTGCTGATTATGGAGATTTAATTAAAGGTACAAGTATATGGGATAATGATTTCACAAAAGATCTGGAACTAAATTTACAAAAAGCGTTTGGAAAACTTTTTCGTAAATATATAAAAAAGAATATTGCTAGTGATGAAAATACTTCATATTCTTCTCTTGATGAATTAAGAGAATCATGGTGGAACACGAACAAAAAATATATTTGGTTAGCAATGAAACATGGTGCGGGAATGAATATTACTACGTGTAATGCTGATGGTAGTGTCACTGGTAGTGGTAGTAGTTGTGATGATATTCCTACGATTGATTTGATCCCGCAATATTTACGGTTTTTGCAAGAATGGGTAGAACATTTTTGCGAACAACGTCAAGCAAAAGTAAAAGATGTGATAAAGAATTGTAATTCGTGTAAGGAAAGTGGAGGTACATGTAATAGTGATTGTGAAAAAAAATGTAAAATAGAGTGTGAAAAATACAAAAAATTTATTGAAGAGTGTCGTACAGCTGCTGAAGGTACTGCCGGATCCTCATGGGTCAAAAGGTGGGACCAAATATATATGAGGTATTCCAAATATATAGAAGACGCGAAACGAAACCGTAAAGCGGGCACAAAAAATTGTGGTACAAGTAGTACTACAAATGCTGCCGCAAGTAAATGTGTACAATCAGATATCGATTCGTTTTTCAAACATTTAATTGATATAGGATTGACCACACCGTCTTCTTATTTATCTATTGTTCTTGATGAAAACAATTGTGGCGCGGACAAAGCTCCTTGGACAACATATACGACATACACAACAACAAAAAATTGTGATATACAAAAAGATAAATCAAAGTCACAATCATGTGATACGCTTGTGGTTGTAAATGTTCCGTCTCCACTGGGTAACACTCCACACGGATATAAATACGCATGCCAGTGTAAAATACCAACTACTGAAGAATCATGTGATGATAGAAAAGAATATATGAACCAGTGGATCATTGATACTAGTAAAAAACAAAAAGGTAGTGGTTCTACAAATAATGATTATGAATTATATACATATAATGGTGTAAAGGAAACAAAGCTACCAAAGAAATCAAGTAGCTCTAAATTAGATGACAAGGATGTGATGTTCTTTAATTTGTTTGAACAGTGGAACAAAGAAATACAATATCAGATAGAGCAGTATATGACAAATACAAAAATATCGTGCAATAACGAGATGAATGTACTGAGTAGTTCATCAGAAGAAAGTAAAAACCAAAATGGTAGTGATCATCACGATGGTAGAAATAACAATACCGATCAGGGTACGAACTGCAAAGAAAAATGTAAATGTTACGAATTATGGATAGAAAAAATTAAAGAACAGTGGGATAAACAAAAGAAGAATTATAATAAATTTCAAAGAAAACAAATATATGATGGAAATAACGCTTCTCATAAAAAAGAAATAGTTAAATTATCTCATTTTTTGTTTTTTTCATGTTGGGAAGAATATATACAAAAATATTTCAATGGCGATTGGAGTAAAATTAAGAATATAGGATCTGATACGTTTGAGTTTCTAATAAAAAAATGTGGAAACGATTCAGGTGATGGAGAAACAATATTTAGTGAAAAATTGAATAATGCACATATAAAATGCAATGAAAATGAAAGTACAGATACTAAAATGAAATCAAGTGAAACATCATCTGTCCTTAACGCAACCAATTATATTCGTGGGTGTCAACCAAAAATTTATGATGGAAAAATATTTCCAGGTAAAGGAGGCGAGAAACAATGGATATGTAAAGATACTATAATACATGGAGATACAAATGGTGCCTGTATCCCTCCAAGAACACAAAATTTATGTGTTGGAAATTTATGGGATAAAAGTTATGGTGGAAGGAGTAATATTAAAAATGATACAAAGGAATCATTAAAACAGAAAATAAAAAATGCTATACAAAAAGAAACAGAATTATTGTATGAATACCACGATAAAGGTACAGCAACCATATCACGAAATCCTATGAAAGGACAAAAAGAAAAAGAAGAAAAAAACAATGATTCTAATGGATTACCAAAAGGTTTTTGTCATGCTGTTCAAAGAAGTTTTATTGATTATAAGAATATGATTTTGGGTACCAGTGTAAATATATATGAGTACATTGGAAAATTACAAGAAGATATAAAAAAAATTATCGAAAAAGGAACAACTAAACAAAACGGAAAAACAGTTGGTAGTGGTGCAGATAAAGTGAACGATTGGTGGAAAGGAATTGAGGGGGAAATGTGGGGTGCCGTAAAATGTGGTATAAAAACAATAAATAAAAAACAAAAGAAGAATGGTACATTTAATGGTAATGAGTGTGGGGTATCCCCCCCAACAGGAAATGATGAGGATCAGTTCGTTTCGTGGTTTAAAGAATGGGGCGAACAGTTTTGTATAGAACGATTACAATATGAAAAAAATATACGTGACGCATGCACTAATAATGGTAAGAATGGAAAGAAATGTATTAATTCAAAAAGTGGTCAAGGAGATAAAATACAAGGAGCATGTAAAAGAAAATGTGAAGAATATAAAAAATATATTTCTGAAAAAAAACAAGAATGGGACAAACAAAAAACAAAATATGAAAATAAATATGTAGGAAAATCTGCGAGTGATTTATTGAAAGAAAATTATCCTGAATGTATATCAGCAAATTTTGATTTTATATTTAACGATAATATTGAATATAAAACATATTATCCATATGGAGATTATAGCAGTATATGTTCGTGCGAACAAGTAAAATATTATGAATATATAAAGGATAAAGGACAAAATAATAAATCTCTTTGTCATGAAAAAGGTAATGATAGGACATGGAGTAAAAAATATATAAAAAAATTGGAAAATGGACGAACATTAGAGGGTGTATACGTCCCCCCAAGACGGCAACAATTATGTCTTTATGAACTATTTCCAATAATTATAAAAAACGAAGAAGGTATGGAAAAGGCAAAAGAAGAATTATTGGAAACATTACAAATAGTTGCAGAGAGAGAAGCATATTATTTATGGAAACAGTATAATCCAACTGGTAAAGGAATTGATGATGCGAATAAGAAAGCTTGTTGTGCTATTCGTGGAAGTTTTTATGATTTGGAAGATATTATTAAAGGCAACGATTTAGTGCATGACGAATACACGAAATATATAGACAGTAAATTAAACGAAATTTTCGGTAGTAGTAATACAAATAATATAGATACAAAACGTGCGCGTACAGATTGGTGGGAAAACGAAACCATTGCTAATGGAACTGATCCTAAAACAATTAGGCAGCTAGTATGGGATGCTATGCAATCTGGAGTAAGATATGCAGTAGAGAAAAAAGAAAATTTTCCTCCATGTATGGGAGTTGAACATATAGGAATAGCCAAACCTCAATTTATAAGATGGTTGGAAGAATGGACAAATGAGTTTTGTGAGAAATATACAAAATATTTCGAAGATATGAAATCCAATTGTGATCCCCCCAAAAGTGCTGATGATTGTGATGATAATAGTAATATTGAATGTAAAAAAGCATGTGCAAATTATGCGAATTGGTTAAATCCAAAAAGGATAGAATGGAATGGAATGAGCAATTATTATAATAAAATATACCGTAAAAGTAACAAAGAATCGGAAGATGGAAAAGATTATTCAATGATTATGGAACCTACAGTCATTGACTATTTGAACAAAAGATGCAATGGCGAAATTAATGGGAACTACATTTGTTGTAGTTGTAAAAATATAGGTGAAAATAGCACTTCACCTGCACAAAAAACAAAAGGATCAAAAAAAAAGGATGAACAATGTGAAGACAATAAAGGACCTCTAGATTTAATGAACGAGGTATTAAATAAAATGGACCCAAAATATAGCAAGTTAAATTTGAAGTGCACAGAAGTTTACTTGGAACATGTTGAAGAACAATTAAAAGAAATTGACAATGCAATAAAAGATTACCAGTTATATCCATTAGATAGATGTTTTGATGATCAGACAAAAATGAAGGTGTGTGATTTAATTCGAGATGCTATAGGATGTAAACATAAGACAAACCTCGATGAACTGGATGAATGGAATGATATGGATATGCGAGGTACTTATAATAAGTATAAAGGTGTTTTAATTCCTCCAAGGCGTAGACAATTATGTTTCTCAAGGATTGTGAGAGGTCGCGCAAATTTAAGAAGCTTAAATGAATTTAAAGAAGAAATTTTAAAAGGAGCCCAATCGGAAGGTAAGTTTTTGGGTAATTATTATAACGAAGATAAAGATAAAGAAAAAAAAGAACATAAAGATAAAGAAAAGGCGCTAGAAGCTATGAAAAACAGTTTTTACGATTATGAAGATATAATAAAAGGTAGTGATATATTAGAAAATATACAATTCAAGGATATTAAAAGGAAATTAGACAAATTACTAACAAAAGAGACTAATAATAATATACAAAACGCTGAAGATTGGTGGAAAGTAAATAATAAATCTATATGGAATGCTATGTTATGTGGGTACAAGAAATCTGGGAATAAAATAATAGATCGATCGTGGTGTACCATACCTACTACAGAAAAAACCCCGCAATTTTTAGGATGGATAAAAGAATGGGGAACAAATGTGTGTATAGAAAAAGAAAAGTATAAAAAAAATGTAAAATCAGAATGTTCGAATGTTACTAATATAGATTTAGATCCACAAGCATCGGAATCAACTAAATGTACATCCGAAATCAGAAAATATCAAGAATGGAGCAGGAAAAGGTCTATTCAGTGGGAAGCTATATCTGAAAGATATAAAAAATATAAGGGTATGGATGAATTTAAAAATGTATTTAACAATGCAAATGAACCGAATGCTAATACATATTTAAAGGAACATTGTTCTAAATGTCCGTGTGGATTTAATGATATGAAAGAAATAACTAAATATACAAACATCGGAAATGAAGCATTTAATACAATAATAGAAAAAGTTAAGATTCCAGCTGAACTTGAAGACGTTATTTACCGGCTAAAACATCATGAGTATGATAAAGGTAATGATTATATTTGTAATAAATATAAAAATATACACGATCGTATGAAAAAAAATAATGGTAATTTTGTGACTGATAATTCCGTTAAAAATTCTTGGGAAATTAATAAAGGTGTGCTAATACCTCCACGAAGAAAAAATTTGTTTCTAAACATTGAGAAATCAGATATATGTGAATATAAAAAAAATCCTAAATTGTTTAAAGATTTCATTTATTGGTCGGCATTTACTGAAGTTGAAAGGTTAAAAATAGTATATCGTCAGGACAAAGAGAAAGTTGCTCATGCAATGAAATATAGTTTTGCCGATATAGGAAATATTATCAAAGGTGATGATATGATGAAAAGCCCGACATCTAAATATATAGAGCAAATATTTAAAGGTACGGAATATAGTGGAATTGATAGTGAAACTTGGTGGGACTCGAATAAATATCATGTATGGGAATCCATGTTATGTGGATACAAAAAAGCCTACGGAAAATTTTCAGAAGATGTTGAAAAAATTCGTGAAATACCTAATAATGATAACGTAAATCAATTTTTACGGTGGTTTACAGAATGGGCACAAGATTTTTGTTATCACCAAGCGGAGGAAATTAAAAAATTAGAGGAAGAATGTAACTTTAATACATGTGAAGAAGCAAATGTTCGTCAAAAATCAGAATGTCAACATCAGTGTAATAAATATAAAAAATTTTTAAGAAAATGGAAAGCTCAATATAACAGACAAAATATTAAATATGAAGGATTAACAGACTCAATTAATATAATAAAAAATAAGGAAGCTCCTAAATTTTTGACGGAACATTGTAAAGAAGAATGCTCATGTTTTCAATCCACAAACGTCAATAATGTTATTAATATGTTTGAAAAATTACCTGATGAGTATATAAAAAAATGTCCTTGTCCAAATGTACCTGAAACATCTTCAACTAAAATAGATGATATCGGGAGTTCAAAACAGAATTCATTTGCAATCCAACATTCAAAGGATAAGAAATTGAATAAATGCGCATTGGATGAAAATATCTGCAAAAATTATGAAGATCATAAATGTAACCCCAAAAAAAACCTTGATGTTCTTGATGAATGGAATAATTTATATCTGGAAGATTTTCAATCTAAAAATAAAGGCGTGCTGATTCCTCCAAGACGAAGACAGTTATGCTTTACGCATATGATTAAAGGTCCTCCCAGAATACAAAATATCGATCAATTTAAGAATGAACTATTAAAAGGTGCTGTTATGGAAGGTAAACGTTTAGGAGAATATTATAAAAATAATAGTGAAAAAGCAATTGAAGCAATGACATACAGTTTTGCTGATTATGCAGATATAATTAAAGGAAATGATATGATAGATACCATACCATTCAAGGATATTAAGCGAAAATTAGAACAAGTTCTTGAACAAGAAGAAAAATCGAACAATGTTCTTAACACAGCAGAACAATGGTGGAAAAAAAATAGAAAACATTTGTGGAATGCGATGTTATGTGGATACAAAAAAACAGGATACATGCCTGATGCATACGTACACCTTTGCATTGTACCCGATACCGATGAAACTCCTCAATTCTTACGATGGATGATAGAATGGGCTAAAACATTCTGTAATGACAAAAGAAATAGAGGAACGTCTATCTTGAAACATTGTAAGGATGAAATTGCTAACAATAAAAACGCTACAAACTCAAGTTATAAATATGAATGCGAAAAGGCTGCTGTGAATTATGTACAATGGGCTAGAAAAATAAATGAAAAATGGACTGGATTATCTGAAAAATTTAAAAGATCCACAAGTTATCTTCCTGATGCATATAAATCATATTCACCTGAACGATATTTAACATCAAAATGTGGTACCTGTGATTGTAAATATAAGGATTTAAAAGAAATAATTGATGCGTACAAGGAAAAAACAATAACAGATAATTTCATTGATACAATTATTGATCAAGCAAAAAACGATAACGAACAAACTCCTTGGTCATGGCTATATCCTTTATCGTGGCCTATGTGGAAAATAGAAGCTGGAATTCCTAAATGGACCATGAAGGGAGATATAACAATTGATTGGCCAGACATTAAATGGCCTAAAATTGACTGGGAAAAACCTGCCTCAAAAGTAAGAGATTCAGTTCACCGAATATCAGATATTATTTTTTATATAATAAACAATACACATATAATACCCACTAAAGATTATAAAGCACATTCCGATTCTTCTGTTACAAATCAGGAAATAAAAGTGTATAAAAATTTAGAAGAACGTAACCTAACTCCCCAATTATATGAACGTCCAGAAATCATCGTTCCTACCATAGGTGCAGTAGCGGCATCTATCATTGGAATCTTACTATACAAGAGAAAACCTAAACATCGACCAAGCAATCTTTTTAGTGTCATTGATATACCTCAAAATGATTATGACATGCCTACAACGAAATCATCAAATAGTTATGTCCCATATGAAAGTGGCCGTTATTCTGGAAAAACATATATTTATATGGAAGGAGACGAAACGGACGATTACAATTATGTTCGTGATATATATTCCTCTGATATTACATCTTCGTCTGAGAGCGAATATGAAGAAGTGGATATCAATGATATATATGTACCAAGTCTTCCCAAATATAAAACGTTCATTGAATTAGTACTAGAACCTTCCAAAAGGGATACATTTAATACATCAAGTGGTGACACATTCACCAATAAACTTACGGATGATGAATGGAACCAATTGAAACAGGATTTTATTGAGCAATATTTAACACATATAGGACCTTCCCTACCATTATATAATGAGTTACCTGATGATAATATGTATATGGATACCCAACCTAATATTTTACATGATAGTATGGATGAAAAACCTTTTATTACTCAAATCCAGGATAGATTTCTTGATAGTAGTCATGAAGAAGTTACTTATAATATTGATTGGAATGTTCCTGAAAATATTAATAGGATTACTAATAACATGGACGATCCAAAATACTCCTCAAATAATATGTATACTGGTACCGATTTAATTAATGATTCATTAAATGGTAACCAATATATTGATATATATGATGAGATGCTGAAACGAAAAGAAAACGAATTATTTGGAACATATCATACAAAATATACAACCTTTAACAGTGTTTCTAAACAAACACCTAGTGACCCGATAATTAACCAACTAGATTTATATCATAAATGGATAGACAAGCATAGAGATATTTGCGAACAGTGGAAAACCAAAGAGGATATGTTATATAAATCGAATGAAGTGTGGAATATGGAACGTAAGGAATATCTATTGGATATACAACCATCAACTCTGGATGATATTCATAAAATTAATGATGAAACATATAATATTATTAGTACAAATAATATATATGATCATCCCTCACAGGAAACCCCCCTCCAACTACTTGGATCAACAAATATTATACCCAGTTATATTACCACGGAACAAAATAATGGATTGCGCACAAATATATCTATGGATACATATATTGATGAAACAAATAATAATAATGTGGTAGCCACTAGTATAATAGGTGACGATCAGATGGAAAATTCGTACAATTGT

>M200101_VAR2CSA_exon1andexon2_protein_seq

MDSKTTIAEKIEEYLKEKSNDSKIDQSLKADPSEVQYYRSGGDGYYLKNNICKITVNHSDSGTNDPCDRKELSYGDNDQWKCDENLSKVSGKPENICVPPRRQRMCIKNLEKLNVDKIRDKHAFLADVLLTARNEGERIILYHPDTNSSNVCVALERSFADLADIIRGRDGNKCETKSPNNVEELIKKFFEKNYRSNEEYKRKYRNDDENYKKLREAWWNANRQKVWEVITCSARSNDLLIKRRWTTSKESNGENKLELFRKCGHYEKEVPTKLDYVPQFLRWLTEWIADLYREKQNLIDDMERHREECTREDDKSKEVTSYCNMCKDKCKKYCECVKKWKTEWENQKNKYNDLYEQENETSQKNTSRYDDYVKEFFEKLNEANYKSLDDYIKGDPYFAEYATKLSFILNSSDANNPSEKIQKNNDEVCNCNESGISSVEQASISDPSSNKTCNTHSSIKTNKKKECKHVKLGVRENDKVLKICVIEDTSLSGVENCCFKDLLGILQEPRIDKNQSGSSSNGSCDKNSEEICQKKLDEALASLHNGYKCDKCKSGTSTVNKNWIWKKFPGNGEGLQKEYANTIGLPPRTHSLYLGNLPKLENVCEDVKDINFDTKEKFLAGCLIAAFHEGKNLKTTYPQNKKKLCKALKYSFADYGDLIKGTSIWDNDFTKDLELNLQKAFGKLFRKYIKKNIASDENTSYSSLDELRESWWNTNKKYIWLAMKHGAGMNITTCNADGSVTGSGSSCDDIPTIDLIPQYLRFLQEWVEHFCEQRQAKVKDVIKNCNSCKESGGTCNSDCEKKCKIECEKYKKFIEECRTAAEGTAGSSWVKRWDQIYMRYSKYIEDAKRNRKAGTKNCGTSSTTNAAASKCVQSDIDSFFKHLIDIGLTTPSSYLSIVLDENNCGADKAPWTTYTTYTTTKNCDIQKDKSKSQSCDTLVVVNVPSPLGNTPHGYKYACQCKIPTTEESCDDRKEYMNQWIIDTSKKQKGSGSTNNDYELYTYNGVKETKLPKKSSSSKLDDKDVMFFNLFEQWNKEIQYQIEQYMTNTKISCNNEMNVLSSSSEESKNQNGSDHHDGRNNNTDQGTNCKEKCKCYELWIEKIKEQWDKQKKNYNKFQRKQIYDGNNASHKKEIVKLSHFLFFSCWEEYIQKYFNGDWSKIKNIGSDTFEFLIKKCGNDSGDGETIFSEKLNNAHIKCNENESTDTKMKSSETSSVLNATNYIRGCQPKIYDGKIFPGKGGEKQWICKDTIIHGDTNGACIPPRTQNLCVGNLWDKSYGGRSNIKNDTKESLKQKIKNAIQKETELLYEYHDKGTATISRNPMKGQKEKEEKNNDSNGLPKGFCHAVQRSFIDYKNMILGTSVNIYEYIGKLQEDIKKIIEKGTTKQNGKTVGSGADKVNDWWKGIEGEMWGAVKCGIKTINKKQKKNGTFNGNECGVSPPTGNDEDQFVSWFKEWGEQFCIERLQYEKNIRDACTNNGKNGKKCINSKSGQGDKIQGACKRKCEEYKKYISEKKQEWDKQKTKYENKYVGKSASDLLKENYPECISANFDFIFNDNIEYKTYYPYGDYSSICSCEQVKYYEYIKDKGQNNKSLCHEKGNDRTWSKKYIKKLENGRTLEGVYVPPRRQQLCLYELFPIIIKNEEGMEKAKEELLETLQIVAEREAYYLWKQYNPTGKGIDDANKKACCAIRGSFYDLEDIIKGNDLVHDEYTKYIDSKLNEIFGSSNTNNIDTKRARTDWWENETIANGTDPKTIRQLVWDAMQSGVRYAVEKKENFPPCMGVEHIGIAKPQFIRWLEEWTNEFCEKYTKYFEDMKSNCDPPKSADDCDDNSNIECKKACANYANWLNPKRIEWNGMSNYYNKIYRKSNKESEDGKDYSMIMEPTVIDYLNKRCNGEINGNYICCSCKNIGENSTSPAQKTKGSKKKDEQCEDNKGPLDLMNEVLNKMDPKYSKLNLKCTEVYLEHVEEQLKEIDNAIKDYQLYPLDRCFDDQTKMKVCDLIRDAIGCKHKTNLDELDEWNDMDMRGTYNKYKGVLIPPRRRQLCFSRIVRGRANLRSLNEFKEEILKGAQSEGKFLGNYYNEDKDKEKKEHKDKEKALEAMKNSFYDYEDIIKGSDILENIQFKDIKRKLDKLLTKETNNNIQNAEDWWKVNNKSIWNAMLCGYKKSGNKIIDRSWCTIPTTEKTPQFLGWIKEWGTNVCIEKEKYKKNVKSECSNVTNIDLDPQASESTKCTSEIRKYQEWSRKRSIQWEAISERYKKYKGMDEFKNVFNNANEPNANTYLKEHCSKCPCGFNDMKEITKYTNIGNEAFNTIIEKVKIPAELEDVIYRLKHHEYDKGNDYICNKYKNIHDRMKKNNGNFVTDNSVKNSWEINKGVLIPPRRKNLFLNIEKSDICEYKKNPKLFKDFIYWSAFTEVERLKIVYRQDKEKVAHAMKYSFADIGNIIKGDDMMKSPTSKYIEQIFKGTEYSGIDSETWWDSNKYHVWESMLCGYKKAYGKFSEDVEKIREIPNNDNVNQFLRWFTEWAQDFCYHQAEEIKKLEEECNFNTCEEANVRQKSECQHQCNKYKKFLRKWKAQYNRQNIKYEGLTDSINIIKNKEAPKFLTEHCKEECSCFQSTNVNNVINMFEKLPDEYIKKCPCPNVPETSSTKIDDIGSSKQNSFAIQHSKDKKLNKCALDENICKNYEDHKCNPKKNLDVLDEWNNLYLEDFQSKNKGVLIPPRRRQLCFTHMIKGPPRIQNIDQFKNELLKGAVMEGKRLGEYYKNNSEKAIEAMTYSFADYADIIKGNDMIDTIPFKDIKRKLEQVLEQEEKSNNVLNTAEQWWKKNRKHLWNAMLCGYKKTGYMPDAYVHLCIVPDTDETPQFLRWMIEWAKTFCNDKRNRGTSILKHCKDEIANNKNATNSSYKYECEKAAVNYVQWARKINEKWTGLSEKFKRSTSYLPDAYKSYSPERYLTSKCGTCDCKYKDLKEIIDAYKEKTITDNFIDTIIDQAKNDNEQTPWSWLYPLSWPMWKIEAGIPKWTMKGDITIDWPDIKWPKIDWEKPASKVRDSVHRISDIIFYIINNTHIIPTKDYKAHSDSSVTNQEIKVYKNLEERNLTPQLYERPEIIVPTIGAVAASIIGILLYKRKPKHRPSNLFSVIDIPQNDYDMPTTKSSNSYVPYESGRYSGKTYIYMEGDETDDYNYVRDIYSSDITSSSESEYEEVDINDIYVPSLPKYKTFIELVLEPSKRDTFNTSSGDTFTNKLTDDEWNQLKQDFIEQYLTHIGPSLPLYNELPDDNMYMDTQPNILHDSMDEKPFITQIQDRFLDSSHEEVTYNIDWNVPENINRITNNMDDPKYSSNNMYTGTDLINDSLNGNQYIDIYDEMLKRKENELFGTYHTKYTTFNSVSKQTPSDPIINQLDLYHKWIDKHRDICEQWKTKEDMLYKSNEVWNMERKEYLLDIQPSTLDDIHKINDETYNIISTNNIYDHPSQETPLQLLGSTNIIPSYITTEQNNGLRTNISMDTYIDETNNNNVVATSIIGDDQMENSYNC

>Mali_PS103_Var2csa_Exon1_DNA_seq

ATGGATAGTACAAGCACTATTGGTGACAAAATTGAAGCATACTTAAAGGAAAAATCCAATGATTCTAAAATAGACCAATCGTTGAAAGCTGACCCTAGTGAAGTGCAGTACTATGGAAGTGGAGGTGATGGATATTACTTAAAAAATAATATTTGCAAAATTACTGTGAATCATTCAGATTCTGGAACAAATGATCCTTGTGAAAAAAAATTACCACCTTATGGCGATAATGACCAATGGAAATGTGACGAAAATTTATATAAAGCAAGTGAGAATAATAAAAATGTATGTGTTCCTCCGAGAAGACAACGTATGTGCATTAACAATTTAGAAAACTTAAAATTTGAAAGAATTAGGGATAAACATGCATTTTTGGCAGATGTATTACTTACGGCCAGAAATGAAGGAGAAAGAATTATACTATATCATCCAGATACAAATAGTTCCAATGTTTGTAATGCGTTAGAAAGAAGTTTTGCTGATCTTGCAGATATTATTAGAGGTAGAGATGAAAACAAGTGTGAAACAAAATCCCCAAATAATGTTGAAGAACTAATAAAAAATTTCTTCGGAAAAAATTACAGGGAGAATAAAGAATATAAAAGAAAATATCGAAATGATGACCAAAAATATACAAAATTACGAGAAGATTGGTGGAATGCTAATAGACAAAAGGTGTGGGAAGTTATTACTTGTAGTGCACGAAGTAATGATTTACTCATAAAACGTCGATGGACAACATCTAAGGAGTCTAATGGAGACAATAAATTGGAATTGTGTCGCAAATGTGGCCATTATGAAGAAAAGGTTCCTACCAAATTAGATTATGTCCCTCAATTCTTAAGGTGGTTAACAGAATGGATAGAGGATTTTTATAGAGAGAAGCAAAATCTGATCGATGACATGGAGAGACACCGTGAAGAGTGTACATCAGAGGATGATAAATCTAAAGAAGTTACATCATATTGTAGTACCTGTAAAGACAAATGTAAGAAATATTGTGAATGTGTGAAGAAATGGAAGACCGAATGGGAGAATCAAAAAAATAAATATAAAGATTTATATCAACAAGAAAACGAAACTTCGCAAAAAAATACATCAAGGTATGATGATTATGTTAAAGAATTTTTTGAAAAACTTAATGAAGCTAATTATAAGTCTCTTGATGATTATATAAAGGATGATCCTTATTTCGCAGAATATGTAACTAAATTATCATTTATTTTAAATTCATCAGATGCTAATACTTCGTCTGGAGAAACAGCAAACCATAATGATGAAGTATGTAACTGTAATGAATCAGAAATTTCATCTGTTGAACAGGAACAAATATCGGGTCCGTCGTCGAATAAAACATGTAACACACATAGCTCTATAAAAGCTAATAAGAAAAAAGTATGTAAACATGTAAAGTTGGGTGTTCGTGAAAAGGATAAAGATTTGAAAATATGCGTAATTGAGGACACTTCCTTAAGTGGTGTTGATAATTGTTGTTTCAAAGATTTATTGGGAATTCTTCAAGAAAATTGTAGTGATAATATACGTGAATCTAGTTCTAATGGTAGTTGTAATAATAATAATGAGGAAATATGTCAAAAGAAATTAGATGAAGCCCTTGCATCTTTAACGAATGGTTATAAATGCGAAAAATGTAAATCTGGAACATCAAGAAGTAAAAAAAATTGGATATGGAAAAAATCCTCTGGTAATGGGGAAGGATTACAAAAAGAATATGCTAATACCATAGGTTTACCCCCAAGAACACATTCGTTATATTTAGGAAATCTACCTAAACTTGAAAATGTGTGCGAAGATGTAACAGATATTAATTTTGATACAAAAGAGAAATTTCTAGCAGGATGCTTAATTGCTGCTTTTCATGAAGGAAAAAATTTAAAAATTTCCAATAAAAATAAAAATGATGACAATAATTCAAAATTATGCAAAGCTTTAGAATATAGTTTTGCCGATTATGGAGATTTAATTAAAGGTACAAGTATATGGGATAATGAATATACAAAAGATCTGGAACTAAATTTACAAAAAATTTTTGGAAAACTTTTTCGTAAATATATAAAAAAGAATAATACTGCTGAACAAGATACTTCATATTCTTCTCTTGATGAATTAAGAGAATCATGGTGGAACACGAACAAAAAATATATTTGGACAGCAATGAAACATGGTGCAGAAATGAATAGTACTACGTGTTGTGGTGATGGTAGTGTCACTGGTAGTAGTGATAGTGGTAGTACTACGTGTAGTGGTGACAATGGTAGTATTAGTTGTGATGATATGTCTACAATTGATTTGATCCCGCAATATTTACGTTTTTTGCAAGAATGGGTAGAACATTTTTGCGAACAACGTCAAGCAAAAGTAAAAGATGTGATAGAGAATTGTAATTCGTGTAAGGAATGTGGAGGTACATGTAACGGTGAGTGTAAAACTGAATGTAAAAATAAATGTAAAGACGAGTGTGAAAAATACAAAAAATTTATTGAAGAGTGTCGTACAGCTGCTGAAGGTACTGCCGAATCCTCATGGGTCAAAAGATGGGACCAAATATATAAGAGGTATTCCAAATATATAGAAGACGCGAAACGAAACCGTAAAGCGGGCACAAAAAATTGTGGTATAACCACTGGCACAATTAGTGGTGAAAGTAGTGGTGCAAACAGTGGCGTAACCACTACCGAAAGTAAATGTGTACAATCAGATATCGATTCGTTTTTCAAACATTTAATTGATATAGGATTGACCACACCATCTTCTTATTTATCTATTGTTCTTGATGAAAACAATTGTGGAGAGGACAATGCTCCGTGGACAACATACACGACATACACGACAACAAAAAATTGTGATATACAAAAAAAAACTCCAAAGTCACAATCATGTAATACTGCCGTGGTTGTAAATGTTCCGTCTCCACTGGGTAACACTCCACACGGATATAAATACGCATGCCAGTGTAAAATACCAACTACTGAAGAATCATGTGATGATAGAAAAGAATATATGAATCAATGGAGTTGTGGTAGCGCACAAACAGTAAGAGGTCGTTCTACAAATAATGATTATGAATTATGTAAATATAATGGTGTAAAGGAAACAAAGCCATTAGGGACATTAAAGAACTCTAAATTAAATGACAAGGATGTGATGTTCTTTAATTTGTTTGAACAGTGGAACAAAGAAATACAATATCAGATAGAGCAGTATATGACAAATACAAAAATATCGTGCAATAACGAGATGAAAGTACTGAGTAGTTCATCAGAAGAAAGTAAAAACCAAAAAGGTAGTGATAATCACGATGGTAGAAATAACAATACCGATCAGGGTACGAATTGCAAAGAAAAATGTGAATGTTACAAATTATGGATAAAAAAAATTAAAGAACAGTGGGATAAACAGAAAGACAATTATAATAAATTTCAAAGAAAACAAATTTATGATGCAAATAAAGGTCCTCAGAATAAAAAAGTTGTTAGTTTATCTAATTTTTTGTTTTTTTCATGTTGGGAAGAATATATACAAAAATATTTCAATGGCGATTGGAGTAAAATTAAGAATATAGGATCTGATACGTTTGATTTTCTAATAAAAAAATGTGGAAACAATTCAGGTGATGGAGAAACAATATTTAATAAAAAATTGAATAATGCAAATATAAAATGTAATGAAAATAAAAGTACCAATAATAAAATGAAATCAAGTGGAAAATCATCTGTCCTTAACGCAACCAATTATATTCGTGGGTGTCAACCAAAAATTTATGATGGAAAAATATTTCCAGGTAAAGGAGGCGAGAAACAATGGATATGTAAAGATACTATAATACATGGAGATACAAATGGTGCCTGTATCCCTCCAAGAACACAAAATTTATGCGTTGGAGAGTTATGGTATAAAAGTTATGGTGGAAGGAGTAATATTAAAAATGATACAAAGGAATCATTAAAAAACAAACTAAAAAATGCTATACAAAAAGAAACGGAATTATTGTATGAATACCACGATAAAGGTACAGCAATTATATCGCAAAATGATAAAAAAGGACAAAAAGGAAAAAATGATCCTAATGGATTGCCAAAAGGTTTTTGTCATGCTGTTCAAAGAAGTTTTATTGATTATAAGAATATGATTTTGGGTACCAGTGTAAATATATATGAATACATTGGAAAATTACAAGAAGATATAAAAAAAATTATCGAAAAAGGAACAACTAAACAAAACGGAAAAACAGTTGGTAGTGGTGCAGAAAACGTAAATGCTTGGTGGAAAGAAATAGAAAAAGATATGTGGGATGCAGTAAGATGTGGTATAAAAACAATAAATAAAAAACAAAAGAAGAATGGTACATTTAGTATCGATGAATGTGGAATATTCCCCCCAACAGGAAATGATGAGGATCAGTTCGTTTCGTGGTTTAAAGAATGGGGCGAACAGTTTTGTATAGAACGATTACAATATGAACAAAATATACGTGACGCATGCACTAATAATGGTAAGAATGGAAAGAAATGTATTAATTCAAAAAATAATAACGAAAAAGAAATACAAGGAGACTGTAAAAGAAAATGTGAAGAATATAAAAAATATATTTCTGAAAAAAAACAAGAATGGGACAAACAAAAAACAAAATATGAAAATAAATATGTAGGAAAATCTGCGAGTGATTTATTGAAAGAAAATTATCCTGAATGTATATCAGCAAATTTTGATTTTATATTTAACGACAAAGCTGATCATAAAAAATATTATCCATATGGAGATTATAGCAGTATATGTTCGTGCGAACAAGTAAAATATTACAAATATAATAATGCTGAGAAAAAAAATAATAAATCTCTTTGTCATGAAAAAGGTAATGATAGGACATGGAGTAAAAAATATATAAAAAAATTGGAAAATGGTCGAACATTAGAGGGTGTATACGTCCCCCCAAGACGGCAACAATTATGTCTTTATGAACTATTTCCAATAATTATAAAAAAAGAAAAGGATATGGAAAAGGCAAAAGAAGAATTATTGGAAACATTACAAATAGTTGCAGAGCGAGAAGCATATTATTTATGGAAACAGTATAATCCAACTGGTAAAGGAATTGATGATGCGAATAAGAAAGCTTGTTGTGCTATTCGTGGAAGTTTTTATGATTTGGAAGATATTATTAAAGGCAACGATTTAGTGCATGACGAATACACGAAATATATAGACAGTAAATTAAATAAAATTTTCGGTAGTAGTAATAAAAATGATATAGATACAAAACGTGCGCGTACAGATTGGTGGGAAAACGAAACAATTGGTGTTGCTAACATAACAGATGCAAATAAAAGTGATAGTAAAACAATTAGGCAGCTAGTATGGGATGCTATGCAATCTGGAGTAAGAAAGGCCATCGATGAAGAAAAGGAAAAAAAAAAACCGAATGAAAATTTTCCTCCATGTATGGGAGTTGAACATATAGGAATAGCCAAACCTCAATTTATAAGATGGTTGGAAGAATGGACAAATGAGTTTTGCGAGAAATATACAAAATATTTTGGAGCTATTAATTCCAATTGTAATCTCAGAAAAGGTGCTGATGATTGTGGTGATAATACTAATATCGAATGTAAAAAAGCATGTGCAAATTATACGAATTGGTTAAATCCAAAAAGGATAGAATGGAATGGAATGAGCAATTATTATAATAAAATATACCGTAAAAGTAACAAAGAATCGGACGATGGAAAAGATTATTCAATGATTATGGAACCTACAGTCATTGACTATTTGAACAAAAGATGCAATGGCGAAATTAATGGGAACTACATTTGTTGTAGTTGTAAAAATATAGGTGAAAATTATAACCCTTCACCTGTAGCGAATAAAAAAGCACAAAGAAAGGAAACAGAATGTGAAGAAGAAAAAGGACCTCTAGATTTAATGAACGAGGTATTAAATAAAATGGACGAGAAATATAGCGAGCACAAGATGAAGTGCACAGAAGTTTACTTGGAACATGTTGAAGAACAATTAAAAGAAATTGACAATGCAATAAAAGATTACCAGTTATATCCATTAGATAGATGTTTTGATGATCAGACAAAAATGAAGGTATGTGATTTAATTGGAGATGCTATAGGATGTAAGGATAAAACAAACCTTGATGAACTTGATGAATGGAATGATATGGACCTGCGAGATCCTTACAATAAGTATAAAGGTGTTTTAATTCCTCCTAGACGTAGACAATTGTGTTTCTCAAGGATTGTGAGAGGTCCCGCTAATTTAAGAAACTTAAATGAATTTAAAGAAGAAATTTTAAAAGGAGCACAATCGGAAGGTAAGTTTTTGGGTAATTATTATAACGAAGATAAAGGTAAAGAAAAAAAAGAAGATCGTAAAGAAAAGGCGCTAGAAGCTATGAAGAACAGTTTTTACGATTATGAATATATAATAAAAGGTACTGATATGTTAGCAAATATACAATTCAAGGATATTAAAAGGAAATTAGACAAATTACTAACAAAAGAGACTAATAATACCAAAAAAGTTGACGATTGGTGGGAAACAAATAAGAAATCTATATGGAATGCTATGTTATGTGGGTACAAGAAATCTGGGAATAAAATAATAGATCCATCATGGTGTACCATACCTACTACAGAAAAAACCCCGCAATTTTTACGATGGATAAAAGAATGGGGAACAAATGTGTGTATACAAAAACAAGAGCATAAAGAATACGTTAAATCAAAATGTTCGAATGTTTCTAATTTAGGGGCACAAGAATCGGAATCAAATAATTGTATACCAGAAATCAGAAAATATCAAGAATGGAGCAGGAAAAGGTATGTTCAGTGGGAAACTATATCGAAAAGATATAAACGTATGGATGAATTTAAAAATGTAAAGGAACCGGATGCTAATGAATATTTGAAGGAACATTGTTCTAAATGTCCGTGTGGATTTAATGATATGAAAGAAATAACTAAATATACAAACATCGGAAATGAAGCATTTAAGCAAATAATAGAACAAGTTGATATTCCAGCTGAACTTGAAGACGTTATTTACCGTCTAAAACATCATGAGTATAACTCGAATGATTATTTTTGTAGGAAATACAATAACAAAAATTTATATTCTCGTATGCAACATAATATTGATACTATTTGGACTGATTTGGTTAAAAATTCTTCGGACATTAATAAAGGTGTGCTAATACCTCCACGAAGAAAAAATTTGTTTCTAAAAATTGATGAATCAGATATATGTAAATATAAAAAAAATCCTAAATTGTTTAAAGATTTCATTTATTCGTCGGCATTTACTGAAGTTGAAAGGTTAAAAATAGTATATCGTCAGGACAAAGAGAAAGTTGCTCATGCAATGAAATATAGTTTTGCCGATATAGGAAATATTATCAAAGGAGATGATATGATGGAAAACAATTCGTCTGATAAGATAGGTAAAATTTTGGGAGGAGATGGAGTCGGACAAAATGAAAAACGTAAAGCTTGGTGGGACATGAATAAATATCATATATGGGAAGCTATGTTATGTGGATACAAACATGCCTACGGAAATATTTCAGAAAATGATAGAAAAATGCTTGATATACCTAATAATGATGACGAACATCAATTTTTACGGTGGTTTACGGAATGGGCAGAAACGTTTTGCTTTCATCAAACAGGAGAATTGGAAAAATTAAAGAAGAACTGTTCATTTTCTAGTTGTGAAACTGCGAAAAATAAAGAAAAAGACAATTGTTTAAATGCTTGTGGGAGTTATAAAAGCTTTCTTAAAAAATGGCAAAAACAATATAAGCAACAATACATTGAATATGAAAATTTAGATAATACAATTCCTGAAAGAAAAGATAAAGGTGCTCATGAATTTTTGAAAGATAAATGCAATAGCAAGTGTTCATGTATTGGTGATAGGGATAATGATTATCTCAATAAGGTTTTCTCAGGAGTACCTTATAAGTATAAAGATAAGTGTCCATGTCCTATAGCATCTGAAAGAACAGAAGTTGTGTCAACTACTACTAAAGAACCAACAGATTGCATAGAAAAATCTGCATATTTATTACAACAAAAGGTAAAAATAGATTCAAAAAATGTTGGGAGTATCTTGAAGAAAACTGCATTATCCACTGATAATTCTTGTAGTCCAGAAAATAAATCTGATAGTTCAACTGATTTTTGTACTTTTAGTGAAAAATATAAAAGTCACAGAAAAATGTTAAATGATACATGTCCCGAAAGGGGAAAAGGATTAATTGCAGGAGAAGAATGGAAATGTAATAACATTAAAAACAAAGAAAGTAATATATGTCTTCCTCCTAGAAGAGAATATATGTGTATAAAAGAATTAGAAGCATTAAATTCTAGTAATGTATACAATAGTGAAACATTGTTAGAAGCAGTTCTAAAAGCAGCAACAAATGAAGGGATTCACATAATAAAGAACTTTGAATCAGAAAATGCATGTAAGCAATACGAAATATGTGATAATATGAAATACAGTTTTGCCGATATAGGTGATATAATTAGAGGGAGAGATTTATGGGATACATCTGAAAATCAAAAAAAAATTCAGAAAACTTTGGAAAATATTTTTAGCAAGATATGTATGAATATCGAAATAAATAAGGTAAAACAAAAATACATGGAACCTCCAAAATATCACAAATTACGTGAAGTATGGTGGGAATTACATAGAGATCAAGTATGGAATGCCATGACACGATGTGCTCCAGAAATAGCTTATCTTCGTAGGAAAGGAATACGTAATAATGAAAATATAACATATTGTGGATATGAAAATTTTACTCCTTATCACGATTATATACCGCAAAGATTAAGATGGATTACAGAATGGAGTGAGTCTTATTGTAAAGCACTAATGAAAAATTTTAATGAATACGAACTAGCATGTAAAACATGTGATATGAATAATGGAAAATGCAATGGAGATTCAGAAGGTAATAAATGTAAAAATTGCAAGGAGAAATGTAAAATATATAAAACTTTTTTGGAAAAATGGAAGGTTCAATTCGAGATACATAAAAAAAAGTTTAAAGACTTGTATGACCAAGTAGAACGACATAATGCTTTTCGGAACAATTTAGTTAGATATCAACAGAAACATGATCGTGATAATAAGTATGTGCAAGATTTTTTAAAGAATATCAAAGATCAATGTAATAATATCGATGGTATTAATATATATCTTGATAGAACAAACCAATGCGAAAATATTACTTTTAAAGAAACACAAATTGCGAATAAAGAATATGCATTTAAATATAAAACAAATGAATATAAGGAAGCCTGTAATTGTATAATACCTGATCCATTAGATAAATGTCCTACACAAGATCACCTGTGCAATTCATTTACATCTATAAGTTCATGTATGAGAAACTCTTTTGGTGATAATCTGGACAAATGGAGTGTAAGAGATGTAACTCATTTTGGAAATAAGAATAAATTTATATTTATTTCTCCAAGAAGACGTAGTTTATGTTTTCAAAATATTAGCTCTTCATATCATCCATTTGAAATAGATAAATTTAAATATAAATTTTTGCAGGGTGCCTTTACTCACGGTAATTGGTTAGCAAAAACATATGGAAATAACGAACAGAAAGCATTGGAAGCAATGAAATATAGTTTTGCTGATTATGCTGATATTATTACAGGAAACGATATTTTTGAATATAGTACTTATAATAAACTAAAAAAGTTATTTCATGATAATGAGATAGGAAGTACTCAAAAATGGTGGAACGATAATAAAAAATCTGTATGGAATGCTATGTTATGTGGATATAAGAAGGGAAATAATTCTTTTGCAATTGATGAAGTAAAATGTGAATTGCCATCTACGGAAGGGATTCCACAATTTCTACGTCGTATAAAAGAATGGGGAACATACTTCTGCGAAGAAAAAGAAAAACAAAAGAATATTGTTAATATAAAATGCACAGATTCTGATCATATAATTGGTAAAAGTATAAAGGCTTCTGAAAAACTAGAATGTAAATTAGCCACTGGGAACTATGAACAGTGGAACACTAATAGATTAAAAGAATGGAAGCTATTATCTCAACAATATAAAACCAATAAAGAATCTAATAAAGATATAGATATGTCTGAATTATCTGCAGAACAATATTTAAAAAAACATTGTATTGGATGTTCATGTCGTTTTTCGGACATGGATGAATTATATATTAAAACTTTAAAAAATGAAAATTTGCATGAAGAAATATTAAAAAACGCTCAGATTCCTATATACTTCGAAGAAGTTTATCATAGATTCGACCGTTCTGATGTTCAGTGCCCTGATAATAATATATGTAATTTATATAAATCTATTCCATGTAAGGAAATGTTAAATGATGAGGAGGTTACAGCTTGGACTACACATTCTGTTACTGATGCATCAAAAGCTATTAAAATTGTACTTATTCCTCCACGAAGAAGAAATTTGTGTTTAGTTATTAATGAAACACAGATGAAAGAATTCAAAACAAATAAAGAAATGTTTAAAAGTTTTATTTATTCATCTGCCGCTTCCGAAGTTACACGATTAAAAAAAGTACATAATGATAATAAAGATAAAGTTCTACAAGCAATGAAATATAGTTTTTCAGATATTGGAAATATTATAAAAGGAGATGATATGATGAAATGTTCAATATCTGATAATATATCTGAAATATTAGATGAAAAAAATGGATCAACCAAAAAACGTATAATTTGGTGGTCACAAAATAAATATCACATATGGGAAGCTATGTTATGTGCATATCAAAAGGATAACAAAAAAATAGAGTTTACTAGTCATGATTGTAAAATGCCTGACACTGAGAAAACTCCTCAATTCTTGAGATGGTTAATTGAATGGTCAAAACAAGTATGTGCACATAAATTATCTAAAGCAAAATATGTAAAAGAAAATTGTTCGAATTATATAAAAGATAAAGAATCTGATAAAGAACAATGTATTAATGCATCAAATATTTATATTCAATGGATGAATAAACAAAAAGAACCATGGAAAATATTATCTAATATTTATACAAACTATAAGAATGAAAAGAATATGAATTACCCAAATCTTCCACGAAATGCTTCATATTATGTAAGAGAAAAATGTCCTGAGTGTATCTGTAATTTGGAACATTTGGAAGAAAAAGAAAATCAGAATCATCAGAATGGTGATCATTTAATAGAAGAAGCAATTAATAAAGCTAAAATGGATTTACCTGACACTGAATGGGGTATATCTTGGGGACCATGGAAAGTTAATTGGGAAAATATGTTTTTTATTAGTTCTTTCTTTCCAAGGATAAAAATAGTTTCTAGAGACACAGCAATAACAAATGATATTTCGAATGTATCTCATTCAAAAGATCCTGTAGCAAAGGATAGATCAATTAATGATGCAATGGTTGATGTAGTAAATATAGTAACTCAACCAGTGACAACCGTAATAGATAATGTTACTCCTACACTTGGTCGTGAACTAAATAAAGTTGTTAATCCCATTAATAATATACTAAATGAAGTTACGACCTCAGTACAAAGTTCAGTAACGAAGGTTAAAGAAACAGGTAATAAAATAACAGAAGATATTAAAAAAATAATTGATAATATGGGAAATATCATGGAAGGTATAATACAATCACTTGAAAAAACAAAAGAAAAAGAACATAAAGAATTTAAATCAAAACAAATATTAGATTCAAAGGAAAATACATATCCAAATACAATTGTACCTACAAATATGGAAGACGTCCTCACGATGTATCATAAAATTGACATTATAGCACCAGCTATTAGCGTTGGAATAATCACTTTAGGATATGCCCTCTACAAGGTAAAA

>Mali_PS103_Var2csa_Exon1_protein_seq

MDSTSTIGDKIEAYLKEKSNDSKIDQSLKADPSEVQYYGSGGDGYYLKNNICKITVNHSDSGTNDPCEKKLPPYGDNDQWKCDENLYKASENNKNVCVPPRRQRMCINNLENLKFERIRDKHAFLADVLLTARNEGERIILYHPDTNSSNVCNALERSFADLADIIRGRDENKCETKSPNNVEELIKNFFGKNYRENKEYKRKYRNDDQKYTKLREDWWNANRQKVWEVITCSARSNDLLIKRRWTTSKESNGDNKLELCRKCGHYEEKVPTKLDYVPQFLRWLTEWIEDFYREKQNLIDDMERHREECTSEDDKSKEVTSYCSTCKDKCKKYCECVKKWKTEWENQKNKYKDLYQQENETSQKNTSRYDDYVKEFFEKLNEANYKSLDDYIKDDPYFAEYVTKLSFILNSSDANTSSGETANHNDEVCNCNESEISSVEQEQISGPSSNKTCNTHSSIKANKKKVCKHVKLGVREKDKDLKICVIEDTSLSGVDNCCFKDLLGILQENCSDNIRESSSNGSCNNNNEEICQKKLDEALASLTNGYKCEKCKSGTSRSKKNWIWKKSSGNGEGLQKEYANTIGLPPRTHSLYLGNLPKLENVCEDVTDINFDTKEKFLAGCLIAAFHEGKNLKISNKNKNDDNNSKLCKALEYSFADYGDLIKGTSIWDNEYTKDLELNLQKIFGKLFRKYIKKNNTAEQDTSYSSLDELRESWWNTNKKYIWTAMKHGAEMNSTTCCGDGSVTGSSDSGSTTCSGDNGSISCDDMSTIDLIPQYLRFLQEWVEHFCEQRQAKVKDVIENCNSCKECGGTCNGECKTECKNKCKDECEKYKKFIEECRTAAEGTAESSWVKRWDQIYKRYSKYIEDAKRNRKAGTKNCGITTGTISGESSGANSGVTTTESKCVQSDIDSFFKHLIDIGLTTPSSYLSIVLDENNCGEDNAPWTTYTTYTTTKNCDIQKKTPKSQSCNTAVVVNVPSPLGNTPHGYKYACQCKIPTTEESCDDRKEYMNQWSCGSAQTVRGRSTNNDYELCKYNGVKETKPLGTLKNSKLNDKDVMFFNLFEQWNKEIQYQIEQYMTNTKISCNNEMKVLSSSSEESKNQKGSDNHDGRNNNTDQGTNCKEKCECYKLWIKKIKEQWDKQKDNYNKFQRKQIYDANKGPQNKKVVSLSNFLFFSCWEEYIQKYFNGDWSKIKNIGSDTFDFLIKKCGNNSGDGETIFNKKLNNANIKCNENKSTNNKMKSSGKSSVLNATNYIRGCQPKIYDGKIFPGKGGEKQWICKDTIIHGDTNGACIPPRTQNLCVGELWYKSYGGRSNIKNDTKESLKNKLKNAIQKETELLYEYHDKGTAIISQNDKKGQKGKNDPNGLPKGFCHAVQRSFIDYKNMILGTSVNIYEYIGKLQEDIKKIIEKGTTKQNGKTVGSGAENVNAWWKEIEKDMWDAVRCGIKTINKKQKKNGTFSIDECGIFPPTGNDEDQFVSWFKEWGEQFCIERLQYEQNIRDACTNNGKNGKKCINSKNNNEKEIQGDCKRKCEEYKKYISEKKQEWDKQKTKYENKYVGKSASDLLKENYPECISANFDFIFNDKADHKKYYPYGDYSSICSCEQVKYYKYNNAEKKNNKSLCHEKGNDRTWSKKYIKKLENGRTLEGVYVPPRRQQLCLYELFPIIIKKEKDMEKAKEELLETLQIVAEREAYYLWKQYNPTGKGIDDANKKACCAIRGSFYDLEDIIKGNDLVHDEYTKYIDSKLNKIFGSSNKNDIDTKRARTDWWENETIGVANITDANKSDSKTIRQLVWDAMQSGVRKAIDEEKEKKKPNENFPPCMGVEHIGIAKPQFIRWLEEWTNEFCEKYTKYFGAINSNCNLRKGADDCGDNTNIECKKACANYTNWLNPKRIEWNGMSNYYNKIYRKSNKESDDGKDYSMIMEPTVIDYLNKRCNGEINGNYICCSCKNIGENYNPSPVANKKAQRKETECEEEKGPLDLMNEVLNKMDEKYSEHKMKCTEVYLEHVEEQLKEIDNAIKDYQLYPLDRCFDDQTKMKVCDLIGDAIGCKDKTNLDELDEWNDMDLRDPYNKYKGVLIPPRRRQLCFSRIVRGPANLRNLNEFKEEILKGAQSEGKFLGNYYNEDKGKEKKEDRKEKALEAMKNSFYDYEYIIKGTDMLANIQFKDIKRKLDKLLTKETNNTKKVDDWWETNKKSIWNAMLCGYKKSGNKIIDPSWCTIPTTEKTPQFLRWIKEWGTNVCIQKQEHKEYVKSKCSNVSNLGAQESESNNCIPEIRKYQEWSRKRYVQWETISKRYKRMDEFKNVKEPDANEYLKEHCSKCPCGFNDMKEITKYTNIGNEAFKQIIEQVDIPAELEDVIYRLKHHEYNSNDYFCRKYNNKNLYSRMQHNIDTIWTDLVKNSSDINKGVLIPPRRKNLFLKIDESDICKYKKNPKLFKDFIYSSAFTEVERLKIVYRQDKEKVAHAMKYSFADIGNIIKGDDMMENNSSDKIGKILGGDGVGQNEKRKAWWDMNKYHIWEAMLCGYKHAYGNISENDRKMLDIPNNDDEHQFLRWFTEWAETFCFHQTGELEKLKKNCSFSSCETAKNKEKDNCLNACGSYKSFLKKWQKQYKQQYIEYENLDNTIPERKDKGAHEFLKDKCNSKCSCIGDRDNDYLNKVFSGVPYKYKDKCPCPIASERTEVVSTTTKEPTDCIEKSAYLLQQKVKIDSKNVGSILKKTALSTDNSCSPENKSDSSTDFCTFSEKYKSHRKMLNDTCPERGKGLIAGEEWKCNNIKNKESNICLPPRREYMCIKELEALNSSNVYNSETLLEAVLKAATNEGIHIIKNFESENACKQYEICDNMKYSFADIGDIIRGRDLWDTSENQKKIQKTLENIFSKICMNIEINKVKQKYMEPPKYHKLREVWWELHRDQVWNAMTRCAPEIAYLRRKGIRNNENITYCGYENFTPYHDYIPQRLRWITEWSESYCKALMKNFNEYELACKTCDMNNGKCNGDSEGNKCKNCKEKCKIYKTFLEKWKVQFEIHKKKFKDLYDQVERHNAFRNNLVRYQQKHDRDNKYVQDFLKNIKDQCNNIDGINIYLDRTNQCENITFKETQIANKEYAFKYKTNEYKEACNCIIPDPLDKCPTQDHLCNSFTSISSCMRNSFGDNLDKWSVRDVTHFGNKNKFIFISPRRRSLCFQNISSSYHPFEIDKFKYKFLQGAFTHGNWLAKTYGNNEQKALEAMKYSFADYADIITGNDIFEYSTYNKLKKLFHDNEIGSTQKWWNDNKKSVWNAMLCGYKKGNNSFAIDEVKCELPSTEGIPQFLRRIKEWGTYFCEEKEKQKNIVNIKCTDSDHIIGKSIKASEKLECKLATGNYEQWNTNRLKEWKLLSQQYKTNKESNKDIDMSELSAEQYLKKHCIGCSCRFSDMDELYIKTLKNENLHEEILKNAQIPIYFEEVYHRFDRSDVQCPDNNICNLYKSIPCKEMLNDEEVTAWTTHSVTDASKAIKIVLIPPRRRNLCLVINETQMKEFKTNKEMFKSFIYSSAASEVTRLKKVHNDNKDKVLQAMKYSFSDIGNIIKGDDMMKCSISDNISEILDEKNGSTKKRIIWWSQNKYHIWEAMLCAYQKDNKKIEFTSHDCKMPDTEKTPQFLRWLIEWSKQVCAHKLSKAKYVKENCSNYIKDKESDKEQCINASNIYIQWMNKQKEPWKILSNIYTNYKNEKNMNYPNLPRNASYYVREKCPECICNLEHLEEKENQNHQNGDHLIEEAINKAKMDLPDTEWGISWGPWKVNWENMFFISSFFPRIKIVSRDTAITNDISNVSHSKDPVAKDRSINDAMVDVVNIVTQPVTTVIDNVTPTLGRELNKVVNPINNILNEVTTSVQSSVTKVKETGNKITEDIKKIIDNMGNIMEGIIQSLEKTKEKEHKEFKSKQILDSKENTYPNTIVPTNMEDVLTMYHKIDIIAPAISVGIITLGYALYKVK

>Mali_PS122_Var2csa_Exon1_DNA_seq

ATGGATAGTAAAACCACTATTGCTAACAAAATTGAAGAATATTTAGAGAAGAAATCCAATGAATCTAATATAGATCAATCGTTGAAAGCTGACCCTAGTGAAGTGCAGTACTATAGAAGTGGAGGTGATGGATATTACTTAAAAAATAATATTTGCAAAATTACTGTGAATCATTCAGATTCTGGAACAAATGATCCTTGTGATAGTATACCACCTCCTTATGGCGATAATGACCAATGGAAATGTCAGCAAAATTCATCTGATGGAAGTGAGAATAATAAAAATATATGTGTTCCTCCGAGAAGACAACGTATGTGCATTAAGAATTTAGAAAAATTAAATGTTGAAAAAATTAGGGATAAACATGCATTTTTGGCAGATGTATTACTTACAGCTAGAAATGAAGGAGAAAGAATTATACTATATCATCCAGATACAAATAGTTCTAATGTTTGTGTTGCGTTAGAAAGAAGTTTTGCTGATCTTGCAGATATTATTAGAGGTAGAGATGGGAACAAGTGTGAAACAAAATCCACAAATAATGTTGAAGAACTAATAAAAAAATTCTTCGGAAAAAATTACAGATCAAATGAAGAATATAGAAAAAAATATCGAAATGATGACCAAAATTATAGAAAATTACGAGAAGATTGGTGGACAAAAAATAGACAAAAGGTGTGGGAAGTTATTACTTGTGGTGCCCGAAGTAACGATTTACTCATAAAACGTCGATGGACAACATCTGGAAAATCTAATGGAGAAAATAAATTGGAATTGTTCCGCAAATGTGGCCATTATGAAGGAAAGGTTCCTACCAAATTAGATTATGTCCCTCAATTTTTAAGGTGGTTAACAGAATGGATAGAGGATTTTTATAGAGAAAAGCAAAATCTGATCGATGACATGGAGAGACACCGTGAAGAGTGTACATCAGAGGATGATAAATGTAAAGAAGGTACATCATATTGTAATATGTGTAAAGACAAATGTAAGAAATATTGTGAATGTGTGAAGAAATGGAAGACCGAATGGGAAAATCAAAAAAATAAATATACAGAATTATATGAACAAGAAAACGAAACTAGCTCTTCACCAAAAAAGAAATCAAGGTATGATGATTATGTTAAAGAATTTTTTGAAAAACTTGAAGCTAATTATAAGTCTCTTGATGATTATATAAAGGGTGATCCTTATTCCAAAGAATATGGAAAACTATTAAAATTTGATAACACTAATGCATTTATTGAAAGTCCAACATATTATAATAATGTATGTTGTTGCAGCACACCTGAAAAATCGGCGAAAACGGATGGATCATCATGTTCAAGTTCTTTTGGAACGCAGGATAACAATTTGTTGGTAGAATTTCAGGAGTTATTGAAAAGTTTTTGCGATACATGTGACACTGATATTGGGGTTGAGGTAGTTTGTGAGAATAATTGCGAAGAGCAATATAAAAAACTGCTCCCCTCTCTTGAGAAATGTACTATTTTGACTTGTAATGAATGCAATAAAACTCGATGTAAACCATTAAAAAAGGTCGAAGAAAAATGGATATGGAAAAAATACTCTGGTAAGGAAGGTGGATTACAAAAAGAATATGCTAATACCATAGGCTTACCCCCAAGAACACAATCCTTATGTTTAGTGTGTTTACATGAAAAAGAAGGAAAAACACAACATAAGACAATTAGCACCAATTCAGAATTATTAAAAGAGAGGATAATTGCTGCTTTTCATGAAGGAAAAAATTTAAAAACTTCCCATGAAAATAAAAATGATGACAATGGAAAAAAATTATGCAAAGCTTTAAAATACAGTTTTGCTGATTATGGAGATTTAATTAAAGGTACAAGTATATGGGATAATGAATATACAAAAGATTTGGAACTAAATTTACAAAAAATTTTTGGAAAACTTTTTCGTAAATATATAAAAAAAAATATTTCTACTGAACAACATACTTTATATTCTTCTCTTGATGAATTAAGAGAATCATGGTGGAACACGAACAAAAAATATATTTGGACAGCAATGAAACACGGTGCAGAAATGAATGGTACTACGTGTTGTGGTGATGGTAGTGTCACTGGTAGTGGTAGTAGTTGTGATGATATTCCTACGATTGATTTGATCCCGCAATATTTACGTTTTTTGCAAGAATGGGTAGAACATTTTTGTAAACAACGTCAAGAAAAAGTAAATGCTGTGATAACGAACTGTAATTCGTGTAAGGAATGTGGAGGTACATGTAACGGTGAGTGTAAAACTGAATGTAAAACTAAATGTAAAGTAGCGTGTGACGCATACAAAGAATTTATTGAAAAGTGTGTTTCAGCTGCTGGTGGTACTTCCGGATCCTCATGGAGCAAAAGGTGGGACCAAATATATAAGAGGTATTCCAAATATATAGAAGACGCGAAACGAAACCGTAAAGCGGGCACCAAAAGTTGTGGTACAAGTAGTACTACAAGTACTGCCGAAAATAAATGTGTACAATCAGATATCGATTCGTTTTTCAAACATTTAATTGATATAGGATTGACCACACCATCTTCTTATTTATCTATTGTTCTTGATGAAAACAATTGTGGAGAGGACAAAGCTCCATGGACAACATACACGACATACACAACAACAAAAAATTGTGATAAAGAAAAAGATAAATCAAAGTCACAATCATGTAATACTGCCGTGGTTGTAAATGTTCCGTCTCCACTGGGTAACACTCCACACGAATATAAATATGTTTGTGAGTGTAGAACACCAAATAAACAAGAATCATGTGATGATAGAAAAGAATATATGAACCAGTGGATCATTGATAACACTAAAAATCCAAAAGGTAGTGGTTCTACAAATAATGATTATGAATTATGTAAATATAATGGTGTAAAGGAAACAAAGCCATTAGGGACATTAAAGAACTCTAAATTAGATGAAAATGATGTGACGTTCTTTAATTTGTTTGAACAGTGGAACAAAGAAATACAATATCAGATAGAGCAGTATATGACAAATACAAAAATATCGTGCAATAACGAGATGAAAGTACTGAGTAGTTCATCAGAAGAAAGTCAAAACCAAAATAGTAGTGATCATCACGATGGTGGAAATAACAATATCAATCAGGGTACGAACTGCAAAAAAAACTGTGAATGTTACAAATTATGGATAGAAAAAATTAAGAAGCAGTGGGGAAAACAGAAAAACAACTACAATAAATTTCGAAGTAAACAAATTTATGATGCAAATAAAGGTCCTCAGAATAAAAAAGTTGTTAGTTTATCTCATTTTTTGTTTTTTTCATGTTGGGAAGAATATATACAAAAACAATTCAATGGAGATTGGAGTAAAATTAAGAATATAGGATCTGATACGTTTGAGTTTTTAATAAAAAAATGTGGAAACAATTCAGGTGATGGAGAAACAATATTTAGTGAAAAATTGAAAAATGCACAACAAAAATGTAAGGAAAATGAAAGTACAGATACTAATATTAATAAAAGTGAAACATCATCTGTCCTTAACGCAACCAATTATATTCGTGGGTGTCAATCAAAAACTTACGATGGAAAAATATTTCCAGGTAAAGGAGGCGAGAAAAAATGGATATGTAAAGATACTATAATACATGGAGATACAAATGGTGCCTGTATCCCTCCAAGAACACAAAATTTATGTGTTGGAAATTTATGGGATAAAAGTTATGGTGGAAGGAGTAATATTAAAAATCATACAAAGGAATCATTAAAAAACAAACTAAAAAATGCTATACAAAAAGAAACGGAATTATTGTATGAATACCACGATAAAGGTACAGCAATTATATCGCAAAATGATAAAAAAGAAAAAGCAAATAATAATAATTCTAATGGATTACCAAAAGGTTTTTGTCATGCTGTTCAAAGAAGTTTTATTGATTATAAGAATATGATTTTGGGTACCAGTGTAAACACATATGAGTACATTGGAAAATTACAAGAAGATATAAAAAAAATTATCGAACAAGAAACAACTAAACAAAACGGAAAAACAGTTGGTAGTGGTGCAGAAAACGTAAATGCTTGGTGGAAAGAAATAGAAAAAGATATGTGGGATGCAGTAAGATGTGGTATAAAAACAATAAATAAAAAAAAGAAGAATGGTACATTTAGTATCGATGAATGTGGAATATTCCCCCCAACAGGAAATGATGAGGATCAGTCCGTTTCGTGGTTTAAAGAATGGGGCGAACAGTTTTGTATAGAACGATTACAATATGAACAAAATATACGTGACGCATGCACTAATAGTGGTCAAGAAGATAAAATACAAGGAGCATGTAAAAGAAAATGTGAAAAATATAAAAAATATATTTCTGAAAAAAAACAAGAATGGGACAAACAAAAAACAAAATATGAAAATAAATATGTAGGAAAATTTGCGAGTGATTTATTGAAAGAAAATTATCCTGAATGTATATCAGCAAATTTTGATTTTATATTTAACGACAAAGCTGATCATAAAAAATATTATCCATATGGAGATTATAGCAGTATATGTTCGTGCGAACAAGTAAAATATTATGAATATAATAAGGATAAAGGACAAAATAATAAATCTCTTTGTCATGAAAAAGGTAATGATAGGACATGGAGTAAAAAATATATAAAAAAATTGGAAAATGGTCGATCATTAGAGGGTGTATACGTCCCCCCAAGACGGCAACAATTATGTCTTTATGAACTATTTCCAATAATTATAAAAAACAAAAATGATATTACAAACGCAAAAGAAGAATTATTGGAAACATTACAAATAGTTGCAGAGAGAGAAGCATATTATTTATGGAAACAGTATCATGCACATAATGATACAACTTATCTTGCACATAAGAAAGCATGTTGTGCTATTCGTGGAAGTTTTTATGATTTGGAAGATATTATTAAAGGCAACGATTTAGTGCATGACGAATACACGAAATATATAGACAGTAAATTAAATAAAATTTTCGGTAGTAGTAATAAAAATGATATAGAGACAAAACGTGCGCGTACAGATTGGTGGGAAAACGAAACATTTGAAGTTTCTAACATAACAGATACAAATAAAAGTGATCGTAAAACAATTAGGCAGCTAGTATGGGATGCTATGCAATCTGGAGTAAGATATGCAGAAGAGAAAAACGAAAATTTTCCTCCATGTATGGGAGTTGAACATATAGGAATAGCCAAACCTCAATTTATAAGATGGTTGGAAGAATGGACAAATGAGTTTTGCGAGAAATATACAAAATATTTCGAAGATATGAAATCCAATTGTAATCTCAGAAAAGGTGCTGATGATTGTGGTGATAATACTAATATCGAATGTAAAAAAGCATGTGCAAATTATACGAATTGGTTAAATCCAAAAAGGATAGAATGGAATGGAATGAGCAATTATTATAATAAAATATACCGTAAAAGTAACAAAGAATCGGAAGATGGAAAAGATTATTCAATGATTATGGAACCTACAGTCATTGACTATTTGAACAAAAGATGCAATGGCGAAATTAATGGGAACTACATTTGTTGTAGTTGTAAAAATATAGGTGAAAATAGCACTTCAGGTACAGTTAATAAAAAACTACAAAAAAAGGAAACAGAATGTGAAGACAATAAAGGACCTCTAGATTTAATGAACGAGGTATTAAATAAAATGGACGAGAAATATAGCGCGCACAAGATGAAGTGCACAGAAGTTTACTTGGAACATGTTGAAGAACAATTAAAAGAAATTGACAATGCAATAAAAGATTACCAGTTATATCCATTAGATAGATGTTTTGATGATCAGACAAAAATGAAGATATGTGATTTAATTGGAGATGCTATAGGATGTAAACATAAGACAAACCTCGAAGAACTTGATGAATGGAATGATATGGACCTGCGAGATCCTTACAATAAGCATAAAGGTGTTTTAATTCCTCCTAGACGTAGACAATTGTGTTTCTCAAGGATTGTGAGAGGTCCCGCAAATTTAAGAAACTTAAATGAATTTAAAGAAGAAATTTTAAAAGGAGCCCAATCGGAAGGTAAGTTTTTGGGTAATTATTATAACGAAGATAAAGGTAAAGAAAAAAAAGAAGATCGTAAAGAAAAGGCGCTAGAAGCTATGAAAAACAGTTTTTACGATTATGAATATATAATAAAAGGTAGTGATATGTTAGCAAATATACAATTCAAGGATATTAAAAGGAAATTAGACAAATTACTAGAAAAAGAGACTAATAATAATACCAAAAAAGCTGAAGATTGGTGGGAAACAAATAAGAAATCTATATGGAATGCTATGTTATGTGGGTACAAGAAATCTGGGAATAAAATAATAGATCCATCATGGTGTACCATACCTACTACAGAAAAAACCCCGCAATTTTTACGATGGATAAAAGAATGGGGAACAAATGTGTGTATAGAAAAAGAAAAGTATAAACAGAATGTAAAATTAGAATGTTCGAATGTTTCTAATTTAGGGGCACAAGAATCGGAATCAAATAATTGTACATCAGAAATTAAAAAATATCAAGAATGGAGCAGGAACAGGTCTATTCAGTGGGAAGCTATATCCGAAAGATATAAAAAATATAAGGGTATGGATGAATTTAAAAATGTATTTAACAATGCAAATGAACCGGATGCTAATACATATTTAAAGGAACATTGTTCTAAATGTCCGTGTGGATTTAATGATATGGAAGAAATAACTAAATATACAAACATCGGAAATGAAGCATTTAAGCAAATAATAGAAAAAGTTAAGATTCCAGCTGAACTTGAAGACGTTATTTACCGAATAAAGCATCATAAGTATGATAAAGGTAATGATTATATTTGTAATAAATATAAAAATATACACGATCGTATGAAAAAAAATAATGGTAATTTTGTGACTGATAATTTCGTTAAAAAATCTTGGGACATTAATAAAGGTGTGCTAATACCTCCACGAAGAAAAAATTTGTTTCTAAACATTGAGAAATCAGATATATGTGAATATAAAAAAAATCCTAAATTGTTTAAAGATTTCATTTATTCGTCGGCATTTACTGAAGTTGAAAGGTTAAAAATAGTATATCGTCAGGACAAAGAGAAAGTTGTTCATGCAATGAAATATAGTTTTGCCGATATAGGAAATATTATCAAAGGAGATGATATGATGGAAAACAATTCGTCTGATAAGATAGGTAAAATTTTGGGAGGAGATGGAGACAGAAAAAATGAAAAACGTAAAAAATGGTGGGACTCGAATAAATATCATATATGGGAATCCATGTTATGTGGATATAAACATGCCTACGGAAATATTTCACACGATGATAAAAAAAAGCTTGATATACCTAATAATGATAACGAACATCAATTTTTACGCTGGTTTACCGAATGGGCCGAAGATTTTTGTCATCACCAAGGGAAAGAATTGAAAACATTACGGGATAAATGTAACTTTGTTAAATGTGACTATGCTACTCCTGAGCAAAAAAGCGCATGCCAACTATCTTGCAACAAATATAAAAAGTTTCTTAACAAATGGGAAGAACAATATATACAACAATATTCTAAATATGACAGTCTTGAAGATACAATTTCTGTAATAACGAAAAAACAACCTTATGAATATTTGAAAGTTAAATGTGGGGATAGGTGTTCTTGTCTTCAAAAAAGAGATGGAATTCCTGTTAATAATGCTTTTGTATATCCACCAAAAGAGGTTGAAGGTAAATGCCCCTGTCCTTTAAAACCTGCTATAATACCCCAAACAGAAACGATTACACCTAGAACTACAAAATCAGAAAAACCTCGTCAATTATACACTGATCACGATTTGAATCAATGTCCTCCAAACCATAATACTTGTAATTATTTTAGTATTTTTGGTACATATAAAAAAAAATATCACCTTGAAGATCTTGATAAATGGAATACACTAGATGTGAAGGATCCTCATGGTACTAATAAAGGTGTGCTAGTTCCTCCAAGACGAAGACAGTTATGCTTTACGCATATGATTAAAGGTCCTCCCAGAATACAAAATATCGATCAATTTAAGAATGAACTATTAAAAGGTGCTGTTATGGAAGGTAAACGTTTAGGAGAATATTATAAAAATAATAGTGAAAAAGCAATTGAAGCAATGACATACAGTTTTGCTGATTATGCAGATATAATTAAAGGAAATGATATGATAGATACCATACCATTCAAGGATATTAAGCGAAAATTAGAACAAGTTCTTGAACAAGAAGAAAAATCGAACAATGTTCTTAACACAGCAGAACAATGGTGGAAAAAAAATAGAAAACATTTGTGGAATGCGATGTTATGTGGATACAAAAAAACAGGATACATGCCTGATGCATACGTACACCTTTGCATTGTACCCGATACCGATGAAACTCCTCAATTCTTACGATGGATGATAGAATGGGCTAAAACATTCTGTAATGACAAAAGAAATAGAGGAACGTCTATCTTGAAACATTGTAAGGATGAAATTGCTAACAATAAAAACGCTACAAACTCAAGTTATAAATATGAATGCGAAAAGGCTGCTGTGAATTATGTACAATGGGCTAGAAAAATAAATGAAAAATGGACTGGATTATCTGAAAAATTTAAAAGATCCACAAGTTATCTTCCTGATGCATATAAATCATATTCACCTGAACGATATTTAACATCAAAATGTGGTACCTGTGATTGTAAATATAAGGATTTACAAGAAATAATTGATGCGTACAAGGAAAAAACAATAACAGATAATTTCATTGATACAATTATTGATCAAGCAAAAAACGATAACGAACAAACTCCTTGGTCATGGCTATATCCTTTATCGTGGCCTATATGGAAAATAGAAGCTGGAATTCCTAAATGGACCATGAAGGGAGATATAACAATTGATTGGCCAGACATTAAATGGCCTAAAATTGACTGGGAAAAACCTGCCTCAAAAGTAAGAGATTCAGTTCACCGAATATCAGATATTATTTTTTATATAATAAACAATACACATATAATACCCACTAAAGATTATAAAGCACATTCCGATTCTTCTGTTACAAATCAGGAAATAAAAGTGTATAAAAATTTAGAAGAACGTAACCTAACTCCCCAATTATATGAACGTCCAGAAATCATCGTTCCTACCATAGGTGCAGTAGCGGCATCTATCATTGGAATCTTACTATACAAGGTATTAGTGTTTGTG

>Mali_PS122_Var2csa_Exon1_protein_seq

MDSKTTIANKIEEYLEKKSNESNIDQSLKADPSEVQYYRSGGDGYYLKNNICKITVNHSDSGTNDPCDSIPPPYGDNDQWKCQQNSSDGSENNKNICVPPRRQRMCIKNLEKLNVEKIRDKHAFLADVLLTARNEGERIILYHPDTNSSNVCVALERSFADLADIIRGRDGNKCETKSTNNVEELIKKFFGKNYRSNEEYRKKYRNDDQNYRKLREDWWTKNRQKVWEVITCGARSNDLLIKRRWTTSGKSNGENKLELFRKCGHYEGKVPTKLDYVPQFLRWLTEWIEDFYREKQNLIDDMERHREECTSEDDKCKEGTSYCNMCKDKCKKYCECVKKWKTEWENQKNKYTELYEQENETSSSPKKKSRYDDYVKEFFEKLEANYKSLDDYIKGDPYSKEYGKLLKFDNTNAFIESPTYYNNVCCCSTPEKSAKTDGSSCSSSFGTQDNNLLVEFQELLKSFCDTCDTDIGVEVVCENNCEEQYKKLLPSLEKCTILTCNECNKTRCKPLKKVEEKWIWKKYSGKEGGLQKEYANTIGLPPRTQSLCLVCLHEKEGKTQHKTISTNSELLKERIIAAFHEGKNLKTSHENKNDDNGKKLCKALKYSFADYGDLIKGTSIWDNEYTKDLELNLQKIFGKLFRKYIKKNISTEQHTLYSSLDELRESWWNTNKKYIWTAMKHGAEMNGTTCCGDGSVTGSGSSCDDIPTIDLIPQYLRFLQEWVEHFCKQRQEKVNAVITNCNSCKECGGTCNGECKTECKTKCKVACDAYKEFIEKCVSAAGGTSGSSWSKRWDQIYKRYSKYIEDAKRNRKAGTKSCGTSSTTSTAENKCVQSDIDSFFKHLIDIGLTTPSSYLSIVLDENNCGEDKAPWTTYTTYTTTKNCDKEKDKSKSQSCNTAVVVNVPSPLGNTPHEYKYVCECRTPNKQESCDDRKEYMNQWIIDNTKNPKGSGSTNNDYELCKYNGVKETKPLGTLKNSKLDENDVTFFNLFEQWNKEIQYQIEQYMTNTKISCNNEMKVLSSSSEESQNQNSSDHHDGGNNNINQGTNCKKNCECYKLWIEKIKKQWGKQKNNYNKFRSKQIYDANKGPQNKKVVSLSHFLFFSCWEEYIQKQFNGDWSKIKNIGSDTFEFLIKKCGNNSGDGETIFSEKLKNAQQKCKENESTDTNINKSETSSVLNATNYIRGCQSKTYDGKIFPGKGGEKKWICKDTIIHGDTNGACIPPRTQNLCVGNLWDKSYGGRSNIKNHTKESLKNKLKNAIQKETELLYEYHDKGTAIISQNDKKEKANNNNSNGLPKGFCHAVQRSFIDYKNMILGTSVNTYEYIGKLQEDIKKIIEQETTKQNGKTVGSGAENVNAWWKEIEKDMWDAVRCGIKTINKKKKNGTFSIDECGIFPPTGNDEDQSVSWFKEWGEQFCIERLQYEQNIRDACTNSGQEDKIQGACKRKCEKYKKYISEKKQEWDKQKTKYENKYVGKFASDLLKENYPECISANFDFIFNDKADHKKYYPYGDYSSICSCEQVKYYEYNKDKGQNNKSLCHEKGNDRTWSKKYIKKLENGRSLEGVYVPPRRQQLCLYELFPIIIKNKNDITNAKEELLETLQIVAEREAYYLWKQYHAHNDTTYLAHKKACCAIRGSFYDLEDIIKGNDLVHDEYTKYIDSKLNKIFGSSNKNDIETKRARTDWWENETFEVSNITDTNKSDRKTIRQLVWDAMQSGVRYAEEKNENFPPCMGVEHIGIAKPQFIRWLEEWTNEFCEKYTKYFEDMKSNCNLRKGADDCGDNTNIECKKACANYTNWLNPKRIEWNGMSNYYNKIYRKSNKESEDGKDYSMIMEPTVIDYLNKRCNGEINGNYICCSCKNIGENSTSGTVNKKLQKKETECEDNKGPLDLMNEVLNKMDEKYSAHKMKCTEVYLEHVEEQLKEIDNAIKDYQLYPLDRCFDDQTKMKICDLIGDAIGCKHKTNLEELDEWNDMDLRDPYNKHKGVLIPPRRRQLCFSRIVRGPANLRNLNEFKEEILKGAQSEGKFLGNYYNEDKGKEKKEDRKEKALEAMKNSFYDYEYIIKGSDMLANIQFKDIKRKLDKLLEKETNNNTKKAEDWWETNKKSIWNAMLCGYKKSGNKIIDPSWCTIPTTEKTPQFLRWIKEWGTNVCIEKEKYKQNVKLECSNVSNLGAQESESNNCTSEIKKYQEWSRNRSIQWEAISERYKKYKGMDEFKNVFNNANEPDANTYLKEHCSKCPCGFNDMEEITKYTNIGNEAFKQIIEKVKIPAELEDVIYRIKHHKYDKGNDYICNKYKNIHDRMKKNNGNFVTDNFVKKSWDINKGVLIPPRRKNLFLNIEKSDICEYKKNPKLFKDFIYSSAFTEVERLKIVYRQDKEKVVHAMKYSFADIGNIIKGDDMMENNSSDKIGKILGGDGDRKNEKRKKWWDSNKYHIWESMLCGYKHAYGNISHDDKKKLDIPNNDNEHQFLRWFTEWAEDFCHHQGKELKTLRDKCNFVKCDYATPEQKSACQLSCNKYKKFLNKWEEQYIQQYSKYDSLEDTISVITKKQPYEYLKVKCGDRCSCLQKRDGIPVNNAFVYPPKEVEGKCPCPLKPAIIPQTETITPRTTKSEKPRQLYTDHDLNQCPPNHNTCNYFSIFGTYKKKYHLEDLDKWNTLDVKDPHGTNKGVLVPPRRRQLCFTHMIKGPPRIQNIDQFKNELLKGAVMEGKRLGEYYKNNSEKAIEAMTYSFADYADIIKGNDMIDTIPFKDIKRKLEQVLEQEEKSNNVLNTAEQWWKKNRKHLWNAMLCGYKKTGYMPDAYVHLCIVPDTDETPQFLRWMIEWAKTFCNDKRNRGTSILKHCKDEIANNKNATNSSYKYECEKAAVNYVQWARKINEKWTGLSEKFKRSTSYLPDAYKSYSPERYLTSKCGTCDCKYKDLQEIIDAYKEKTITDNFIDTIIDQAKNDNEQTPWSWLYPLSWPIWKIEAGIPKWTMKGDITIDWPDIKWPKIDWEKPASKVRDSVHRISDIIFYIINNTHIIPTKDYKAHSDSSVTNQEIKVYKNLEERNLTPQLYERPEIIVPTIGAVAASIIGILLYKVLVFV

>C0111a01_Var2csa_Exon1_DNA_seq

ATGGATAGTACAAGCACTATTGCTAACAAAATTGAAGAATATTTAGGTGCAAAATCCAATGATTCTAAAATAGACGAATTGTTGAAAGCTGACCCTAGCGAGATAGACTATTATAATTTTGGAGGTGATGGAGATTACTTAAAAAATAATATTTGTAAAATTACCGTGAATCATTCAGATTCTGGAAAGTATGATCCTTGTGAAAAAAAATTACCACCTTATGATGATAATGACCAATGGAAATGTCAGCAAAATTCATCTGATGGAAGTGAAAAACCTGAAAATATATGTGTCCCTCCGAGAAGACAACGTATGTGCATTAACAATTTAGAAAACTTAAAATTTGATAAAATTAGGGATAATAATGCATTTTTGGCAGATGTATTACTTACAGCTAGAAATGAAGGAGAAAAAATAGTGCAGAATCATCCAGATACAAATAGTTCTAATGTTTGTGTTGCGTTAGAAAGAAGTTTTGCTGATCTTGCAGATATTATTAGAGGTACAGATCAATTGAAAGGTTCTAACATTTTAGAACAAAATTTAAGACGAATGTTTAAGAATATACTAGAAAAAGGCAGCACAATTCAAAGTAATTATTCAAAGGACCAAAATTATAGAAAATTACGAGAAGATTGGTGGACAAAAAATAGACAAAAGGTGTGGGAAGTTATTACTTGCGGTGCACGAAGTAACGATTTACTCATAAAACGTCGATGGAGAACATCTAAGGAGTCTAATGGAGAAAATAAATTGGAATTGTTCCGCAAATGTGGCCATTATGAAGGAAAGGTTCCTACCAAATTAGATTATGTCCCTCAATTTTTAAGGTGGTTAACAGAATGGATAGAGGATTTTTATAGAGAAAAGCAAAATCTGATCGATGACATGGAGAGACACCGTGAAGAGTGTACATCAGAGGATGATAAATCTAAAGAAGTTACATCATATTGTAGTACCTGTAAAGACAAATGTAAGAAATATTGTGAATGTGTGAAGAAATGGAAGACCGAATGGGAGAATCAAAAAAATAAATATACAGAATTATATGAACAAAACAAAAACAAAACTTCGCAAAAAAATACATCAAGGTATGATGATTATGTTAAAGAATTTTTTGAAAAACTTAAAGGAAATGGATATTCCTCTGCCAACAGGGTT

>C0111a01_Var2csa_Exon1_protein_seq

MDSTSTIANKIEEYLGAKSNDSKIDELLKADPSEIDYYNFGGDGDYLKNNICKITVNHSDSGKYDPCEKKLPPYDDNDQWKCQQNSSDGSEKPENICVPPRRQRMCINNLENLKFDKIRDNNAFLADVLLTARNEGEKIVQNHPDTNSSNVCVALERSFADLADIIRGTDQLKGSNILEQNLRRMFKNILEKGSTIQSNYSKDQNYRKLREDWWTKNRQKVWEVITCGARSNDLLIKRRWRTSKESNGENKLELFRKCGHYEGKVPTKLDYVPQFLRWLTEWIEDFYREKQNLIDDMERHREECTSEDDKSKEVTSYCSTCKDKCKKYCECVKKWKTEWENQKNKYTELYEQNKNKTSQKNTSRYDDYVKEFFEKLKGNGYSSANRV
