# Supplementary material for: Placental malaria vaccine candidate antigen VAR2CSA displays atypical domain architecture in some Plasmodium falciparum strains
Source: Commun Biol. 2019 Dec 6;2:457. doi: 10.1038/s42003-019-0704-z (PMC6897902; doi:10.1038/s42003-019-0704-z)
Supplement: Supplementary file 8 — Reporting Summary [file 42003_2019_704_MOESM8_ESM.pdf]

nature research

Corresponding author(s): Patrick E. Duffy

Last updated by author(s): Nov 8, 2019

Reporting Summary

Nature Research wishes to improve the reproducibility of the work that we publish. This form provides structure for consistency and transparency in reporting. For further information on Nature Research policies, see Authors & Referees and the Editorial Policy Checklist.

Statistics

For all statistical analyses, confirm that the following items are present in the figure legend, table legend, main text, or Methods section.

n/a	Confirmed	
☒	☐	The exact sample size (*n*) for each experimental group/condition, given as a discrete number and unit of measurement
☒	☐	A statement on whether measurements were taken from distinct samples or whether the same sample was measured repeatedly
☒	☐	The statistical test(s) used AND whether they are one- or two-sided<br>*Only common tests should be described solely by name; describe more complex techniques in the Methods section.*
☒	☐	A description of all covariates tested
☒	☐	A description of any assumptions or corrections, such as tests of normality and adjustment for multiple comparisons
☒	☐	A full description of the statistical parameters including central tendency (e.g. means) or other basic estimates (e.g. regression coefficient) AND variation (e.g. standard deviation) or associated estimates of uncertainty (e.g. confidence intervals)
☒	☐	For null hypothesis testing, the test statistic (e.g. *F*, *t*, *r*) with confidence intervals, effect sizes, degrees of freedom and *P* value noted<br>*Give P values as exact values whenever suitable.*
☒	☐	For Bayesian analysis, information on the choice of priors and Markov chain Monte Carlo settings
☒	☐	For hierarchical and complex designs, identification of the appropriate level for tests and full reporting of outcomes
☒	☐	Estimates of effect sizes (e.g. Cohen's *d*, Pearson's *r*), indicating how they were calculated

Our web collection on statistics for biologists contains articles on many of the points above.

Software and code

Policy information about availability of computer code

Data collection	The pooled libraries were sequenced on a HiSeq 4000 (Illumina). The data was processed using RTA version 2.7.7 and CASAVA 1.8.2.
Data analysis	Raw Illumina FASTQ data aligned by Bowtie2 (version 2.3.4.3). BAM pileups converted to consensus DNA sequences by SAMTOOLS (version 1.9). Aligned and unaligned Illumina reads converted to consensus protein sequences by DuffyNGS (version 1.8, available on GitHub).

For manuscripts utilizing custom algorithms or software that are central to the research but not yet described in published literature, software must be made available to editors/reviewers. We strongly encourage code deposition in a community repository (e.g. GitHub). See the Nature Research guidelines for submitting code & software for further information.

Data

Policy information about availability of data

All manuscripts must include a data availability statement. This statement should provide the following information, where applicable:

- Accession codes, unique identifiers, or web links for publicly available datasets
- A list of figures that have associated raw data
- A description of any restrictions on data availability

The raw FASTQ sequences of M200101 (SRR8176247) presented in this article have been submitted to the Gene Expression Omnibus (www.ncbi.nlm.nih.gov/geo) under project # PRJNA504638. The full-length DNA and amino acids sequences of VAR2CSA from isolates M200101, Mali_PS103, Mali_PS122 and C0111a0 are shown in Supplementary Data 4 and submitted to GenBank (accession number MN631060 - MN631063).

Field-specific reporting

Please select the one below that is the best fit for your research. If you are not sure, read the appropriate sections before making your selection.

☒ Life sciences ☐ Behavioural & social sciences ☐ Ecological, evolutionary & environmental sciences

For a reference copy of the document with all sections, see nature.com/documents/nr-reporting-summary-flat.pdf

Life sciences study design

All studies must disclose on these points even when the disclosure is negative.

Sample size	No sample-size calculation was performed for this work.
Data exclusions	RNA-seq samples with limited read depth across var2csa to return a full-length protein sequence were removed from the Consensus Protein Pileups analysis.
Replication	Replicate experiments were successful.
Randomization	No randomization was performed. Samples analyzed in this work were selected from study surveying binding phenotypes of field isolates collected from children and pregnant women in Mali. RNA-seq data was generated and analyzed for var2csa transcription by the isolates at different timepoints during adaptation to long-term culture. DNA-seq and RNA-seq data from public databases were also analyzed to define variation in VAR2CSA domain structure.
Blinding	Blinding is not relevant to this study as there was no group allocation done

Reporting for specific materials, systems and methods

We require information from authors about some types of materials, experimental systems and methods used in many studies. Here, indicate whether each material, system or method listed is relevant to your study. If you are not sure if a list item applies to your research, read the appropriate section before selecting a response.

Materials & experimental systems

n/a	Involved in the study	
☐	☒	Antibodies
☐	☒	Eukaryotic cell lines
☒	☐	Palaeontology
☒	☐	Animals and other organisms
☐	☒	Human research participants
☒	☐	Clinical data

Methods

n/a	Involved in the study	
☒	☐	ChIP-seq
☐	☒	Flow cytometry
☒	☐	MRI-based neuroimaging

Antibodies

Antibodies used	Goat anti-Human IgG Fc Secondary Antibody, PE, eBioscience™from Invitrogen by Thermo Fischer Scientific; Cat # 12-4998-82; Lot # 1962116
Validation	From the manufacturer's website (https://www.thermofisher.com/antibody/product/Goat-anti-Human-IgG-Fc-Secondary-Antibody-Polyclonal/12-4998-82)<br>Description: This goat antibody reacts with the Fc fragment of heavy chains on human IgG but not with the light chains on most human immunoglobulins. No antibody was detected against human IgM or IgA, or against nonimmunoglobulin serum proteins. This antibody has been tested to ensure minimal cross-reaction with bovine, horse, and mouse serum proteins, but the antibody may cross-react with immunoglobulins from other species.<br><br>Applications Reported: PE goat anti-Human IgG (Fc gamma Fragment Specific) has been reported for use in flow cytometric analysis.<br><br>Applications Tested: PE goat anti-Human IgG (Fc gamma Fragment Specific) has been tested by flow cytometric analysis to detect purified human Fc gamma-tagged recombinant proteins. This can be used at less than or equal to 1 µg per test. A test is defined as the amount (µg) of antibody that will stain a cell sample in a final volume of 100 µL. Cell number should be determined empirically but can range from 10^5 to 10^8 cells/test. It is recommended that the antibody be carefully titrated for optimal performance in the assay of interest.<br><br>Excitation: 488-561 nm; Emission: 578 nm; Laser: Blue Laser, Green Laser, Yellow-Green Laser.

Filtration: 0.2 µm post-manufacturing filtered.

Eukaryotic cell lines

Policy information about cell lines

Cell line source(s)	Expi293F cells from ThermoFisher
Authentication	Cells are commercially available and authenticated according to the manufacturer. (see https://www.thermofisher.com/order/catalog/product/A14527?SID=srch-srp-A14527)
Mycoplasma contamination	Cells lines were not tested for mycoplasma contamination during the expression of the recombinant proteins used in this work. The cell stocks are purchased from ThermoFisher as mycoplasma free and our stocks are made from early passage from purchased stock. Mycoplasma contamination test performed for cultures in the lab were negatives.
Commonly misidentified lines (See ICLAC register)	None

Human research participants

Policy information about studies involving human research participants

Population characteristics	Briefly, pregnant women were enrolled between November 2010 and October 2013 into a longitudinal cohort study of mother-infant pairs conducted in Ouélessébougou, Mali. The study site is located 80 km south of Bamako, an area of intense seasonal malaria transmission during the rainy season from July to December.
Recruitment	Pregnant women aged 15–45 years without clinical evidence of chronic or debilitating illness were asked to participate in the study and gave signed informed consent after receiving a study explanation form and oral explanation from a study clinician in their native language.
Ethics oversight	The protocol and study procedures were approved by the institutional review board of the National Institute of Allergy and Infectious Diseases at the US National Institutes of Health, and the Ethics Committee of the Faculty of Medicine, Pharmacy and Dentistry at the University of Bamako, Mali.

Note that full information on the approval of the study protocol must also be provided in the manuscript.

Flow Cytometry

Plots

Confirm that:

- [x] The axis labels state the marker and fluorochrome used (e.g. CD4-FITC).
- [x] The axis scales are clearly visible. Include numbers along axes only for bottom left plot of group (a 'group' is an analysis of identical markers).
- [x] All plots are contour plots with outliers or pseudocolor plots.
- [x] A numerical value for number of cells or percentage (with statistics) is provided.

Methodology

Sample preparation	M200101 infected erythrocytes at the trophozoite stage were incubated with PAM1.4 or a negative control monoclonal (MPE8) and washed. IE were labeled with 0.1% SYBR green while bound antibodies were stained with PE-conjugated anti-human IgG.
Instrument	LSRII
Software	BD FACSDIVA version 8.0 and FlowJo version 10
Cell population abundance	50000 labeled cells with 8.2% of infected erythrocytes were acquired and anylyzed for surface expression of VAR2CSA.
Gating strategy	From FSC/SSC gate, cells were gated to FITC and PE. Positives cells expressing VAR2CSA were defined as FITC+PE+

- [ ] Tick this box to confirm that a figure exemplifying the gating strategy is provided in the Supplementary Information.
